# Minimally invasive approaches versus conventional sternotomy for aortic valve replacement in patients with aortic valve disease: a systematic review and meta-analysis of 17 269 patients

**DOI:** 10.1097/MS9.0000000000002204

**Published:** 2024-06-04

**Authors:** Saad Khalid, Muhammad Hassan, Abraish Ali, Farah Anwar, Mishal Shan Siddiqui, Sunita Shrestha

**Affiliations:** a Department of Medicine, Dow University of Health Sciences, Karachi, Pakistan; b Upendra Devkota Memorial National Institute of Neurological and Allied Sciences Bansbari, Kathmandu, Nepal

**Keywords:** aortic valve replacement, full sternotomy, mini-sternotomy, right mini-thoracotomy

## Abstract

**Background:**

Aortic valve replacement (AVR) is a common procedure for aortic valve pathologies, particularly in the elderly. While traditional open AVR is established, minimally invasive techniques aim to reduce morbidity and enhance treatment outcomes. The authors’ meta-analysis compares these approaches with conventional sternotomy, offering insights into short and long-term mortality and postoperative results. This study provides valuable evidence for informed decision-making between conventional and minimally invasive approaches for AVR.

**Materials and methods:**

Till August 2023, PubMed, Embase, and MEDLINE databases were searched for randomized controlled trials (RCT) and propensity score matched (PSM) studies comparing minimally invasive approaches [mini-sternotomy (MS) and right mini-thoracotomy (RMT)] with full sternotomy (FS) for AVR. Various outcomes were analyzed, including mortality rates, bypass and clamp times, length of hospital stay, and complications. Risk ratios (RR) and the weighted mean differences (WMD) with corresponding 95% CIs were calculated using Review Manager.

**Results:**

Forty-eight studies were included having 17 269 patients in total. When compared to FS, there was no statistically significant difference in in-hospital mortality in MS (RR:0.80; 95% CI:0.50–1.27; I^2^=1%; *P*=0.42) and RMT (RR:0.70; 95% CI:0.36–1.35; I^2^=0%; *P*=0.29). FS was also linked with considerably longer cardiopulmonary bypass duration than MS (MD:8.68; 95% CI:5.81–11.56; I^2^=92%; *P*=0.00001). The hospital length of stay was determined to be shorter in MS (MD:−0.58; 95% CI:−1.08 to −0.09; I^2^=89%; *P*=0.02) with no statistically significant difference in RMT (MD:−0.67; 95% CI:−1.42 to 0.08; I^2^=84%; *P*=0.08) when compared to FS.

**Conclusions:**

While mortality rates were comparable in minimally invasive approaches and FS, analysis shows that MS, due to fewer respiratory and renal insufficiencies, as well as shorter hospital and ICU stay, may be a safer approach than both RMT and FS.

## Introduction

Highlights

- Minimally invasive techniques for aortic valve replacement (AVR), which include mini-sternotomy (MS) and right mini-thoracotomy (RMT) have enhanced treatment outcomes, reduced complications, but their effectiveness remains inconclusive.
- We analyzed various outcomes, including mortality rates, bypass and clamp times, length of hospital and ICU stay, and complications.
- Our pooled analysis shows that MS may be a safer approach than both RMT and full sternotomy (FS). This is likely due fewer respiratory and renal insufficiencies, as well as shorter hospital and ICU stay.

Aortic valve replacement (AVR) is a frequently performed procedure due to the high burden of aortic valve pathologies, especially in individuals with advancing ages^1^. While the traditional open technique has demonstrated favorable postoperative outcomes^2^, the increasing adoption of minimally invasive surgery has led to innovations in AVR. These techniques were developed to reduce the morbidity and complication rates associated with the traditional AVR, and are also considered beneficial, especially for frailer, older patients^3,4^.

The conventional AVR is performed via a midline sternotomy providing maximal exposure to the mediastinum. On the other hand, techniques aimed at reducing the incision size include partial upper or lower sternotomies, anterior thoracotomies, and transverse sternotomies^5^. Of these, partial upper sternotomy, also referred to as mini-sternotomy, is the most widely employed^5^. Such approaches to the aortic valve are presumed to expedite postoperative recovery by reducing hospital stay, decreasing bleeding and wound infection, and minimizing postoperative pain^6,7^. However, mixed results regarding these outcomes have been reported across literature. While minimally invasive interventions are invariably associated with better cosmesis, data surrounding their efficacy and long-term mortality has also been inconclusive^6^.

A meta-analysis of 42 studies published by Ogami *et al.* ^8^ in 2022 demonstrated improved operative mortality and decreased in-hospital stay with mini-sternotomy (MS) when compared to full sternotomy (FS) with equivalent midterm mortality rates. Herein, we present an updated systematic review and meta-analysis of 48 studies including Randomized Controlled Trials (RCTs) and propensity matched observational studies to compare the aforementioned techniques including a comparison with right mini-thoracotomy (RMT) for short and long-term mortality and postoperative outcomes.

## Methods

### Literature search and study selection

In this meta-analysis, we complied with Preferred Reporting Items for Systematic Reviews and Meta-Analysis (PRISMA, Supplemental Digital Content 3, http://links.lww.com/MS9/A500) 2020 guidelines^9^. In addition, this meta-analysis is reported in line with A Measurement Tool to Assess systematic Reviews 2 (AMSTAR 2, Supplemental Digital Content 4, http://links.lww.com/MS9/A501) guidelines^10^. We registered this protocol with Prospero (CRD42023453991). A systematic search of databases was conducted from the inception till August, 2023, on PubMed/MEDLINE, Embase, clinicaltrials.gov and Cochrane CENTRAL library. Several MeSH terms were used: (mini sternotomy OR hemi sternotomy OR hemi upper sternotomy OR right anterior mini thoracotomy OR mini thoracotomy OR right mini thoracotomy OR anterior mini thoracotomy) AND (full sternotomy OR full median sternotomy OR median sternotomy) AND (aortic valve repair OR Transcatheter Aortic Valve Implantation OR aortic valve replacement OR aortic valve implantation). Only articles written in English language were included. The extracted studies were carefully reviewed by two independent authors (SK and AA). Abstracts or full manuscripts when necessary were screened to assess whether a study met the eligibility criteria. The studies included in this meta-analysis were both RCTs and observational studies. SK and AA searched the databases and retrieved the articles, and MS was consulted in case of any discrepancy. The articles were initially selected by reading the title and the abstract. Finally, a full-text review was conducted, and relevant articles that met the inclusion and exclusion criteria were selected.

### Eligibility criteria

Studies were included based on the following eligibility criteria: (1) Observational studies with propensity matching and RCTs, (2) studies reporting the outcome comparisons between two of MS, RMT, and FS approaches and (3) only articles written in English language were included. Studies with combined data for minimally invasive approaches were excluded and those that were subsets of other studies to avoid data duplication. Review articles, editorials, case reports, non-comparative studies and study protocols were excluded. Grey unpublished literature, references of relevant meta-analyses, and review articles were also screened for potential studies.

### Data extraction and quality assessment

The data that were extracted included name of authors, year of study, study design, country in which the study was conducted, mortality outcomes like one year mortality, in-hospital mortality, 30-day mortality, operative mortality, surgical site infection, stroke, transient ischemic attack, cross-clamp time, operative time, Cardiopulmonary bypass (CPB) time, hospital length of stay, ICU stay, ventilation time, 30-day readmissions, extracorporeal membrane oxygenation, low cardiac output, reoperation for bleeding, major bleeding event, red cell transfusion, drainage bleed per 24 h, postoperative atrial fibrillation, permanent pacemaker, postoperative intra-aortic balloon pump (IABP), postoperative myocardial infarction (MI), complete atrioventricular (AV) block, pneumonia, paravalvular leaks, sternum re-fixation, respiratory insufficiency, respiratory failure, tracheostomy, pleural effusion requiring puncture, new onset dialysis, renal failure, renal insufficiency, sepsis, postoperative confusion and gastrointestinal complications. The risk assessment in individual studies was evaluated by two independent authors (S.K. and A.A.) using the Cochrane Risk of Bias tool in RCTs^11^ and the Newcastle‐Ottawa Scale for propensity score matched (PSM) studies^12^.

### Data analysis

Review Manager v.5.4 was used for all statistical analyses. Mean duration with standard deviation was extracted. Dichotomous outcomes were pooled as relative risks (RRs) with 95% CIs. Continuous outcomes were presented as the weighted mean difference (MD) with 95% CI. When the median and interquartile or overall range was reported, the mean values and standard deviations (SDs) were estimated as proposed by Luo *et al.* ^13^. and Wan *et al.* ^14^. Studies were divided into two subgroups namely MS versus FS and/or RMT versus FS. Random effects model was used to assess the treatment effect to account for the inter‐study variability and to construct forest plot. Heterogeneity was considered to be present when the *I* ^*2*^ index was greater than 50%. Publication bias was assessed using funnel plots. A *P* value of less than 0.05 was considered statistically significant.

## Results

### Literature search and quality assessment

**Figure 1:**
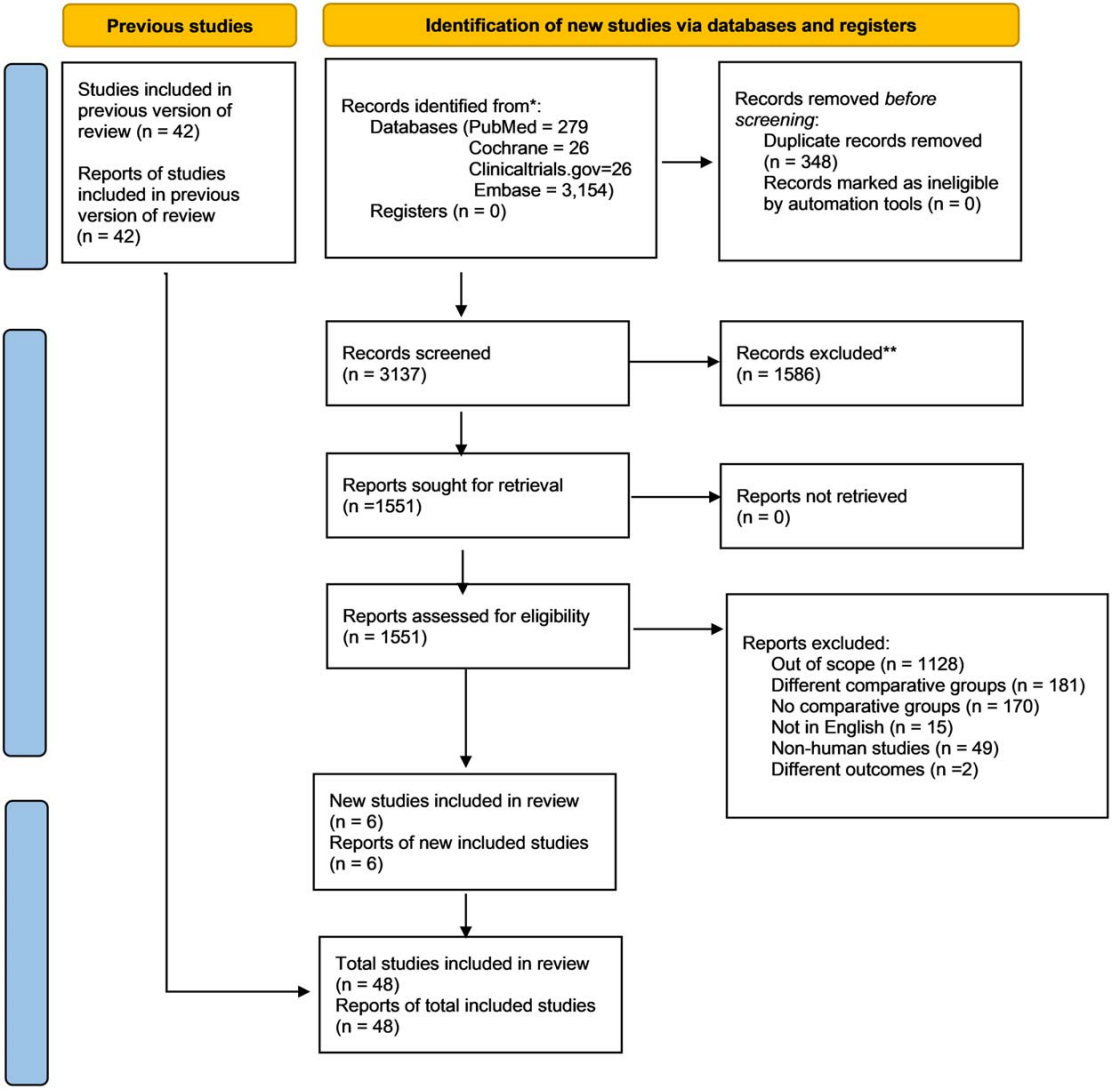
PRISMA flow diagram. PRISMA, Preferred Reporting Items for Systematic Reviews and Meta-Analysis.

The initial literature search identified 3485 articles. After a detailed evaluation of these articles according to the inclusion criteria, 48 studies were selected for analysis which included 15 RCTs and 33 observational studies involving a total of 9923 patients in the minimally invasive group and 7346 in the FS group. Two types of Minimally invasive aortic valve replacement (MIAVR) interventions were included in the analysis where 36 studies included MS, 7 RMT and 5 both. The PRISMA flow chart (Fig. 1) summarizes results of our literature search. Supplemental Digital Content (SDC) 1, Table S1, http://links.lww.com/MS9/A498 outlines the baseline characteristics of all included studies.

### Risk assessment and publication bias

Quality assessment using the Cochrane risk of bias assessment tool for RCTs demonstrated eight high-risk studies, six moderate risk and one low risk study (SDC 2, Figure S1, S2, Supplemental Digital Content 2, http://links.lww.com/MS9/A499). Newcastle-Ottawa scores for observational studies indicated low risk of bias in majority of the included studies (SDC 1, Table S2, Supplemental Digital Content 1, http://links.lww.com/MS9/A498). Funnels plots for publication bias are illustrated in SDC 2, Figure S3-S19, Supplemental Digital Content 2, http://links.lww.com/MS9/A499.

### Mortality outcomes

**Figure 2:**
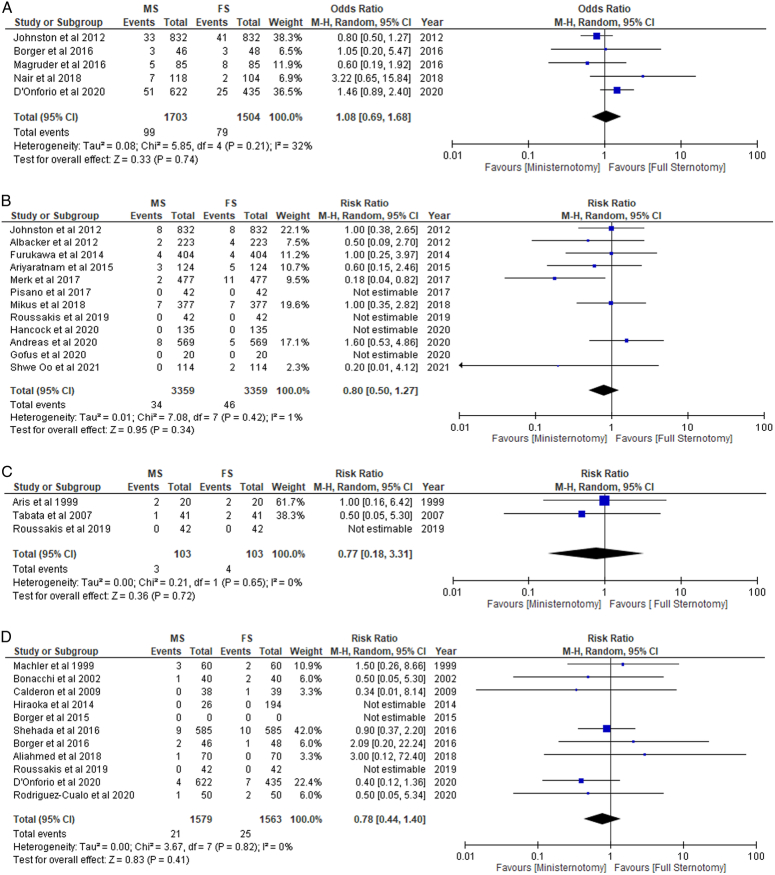
(A) 1-year mortality. (B) In-hospital mortality. (C) Operative mortality. (D) 30-day hospitality in patients undergoing mini-sternotomy versus full sternotomy. FS, full sternotomy; M-H, Mantel-Haenszel; MS, mini-sternotomy.

**Figure 3:**
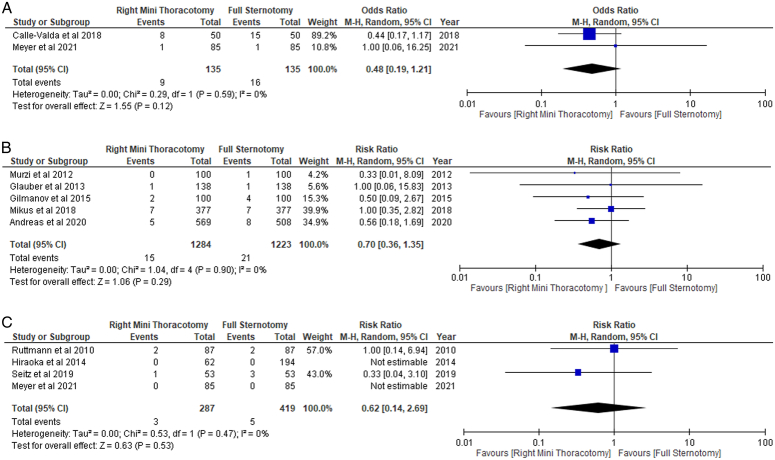
(A) 1-year mortality. (B) In-hospital mortality. (C) 30-day mortality in patients undergoing right mini-thoracotomy versus full sternotomy. M-H, Mantel-Haenszel.

In patients undergoing MS, outcomes such as 30 days, operative and in-hospital mortality occurred less frequently, whereas 1-year mortality occurred more frequently when compared to FS (RR: 1.07; 95% CI: 0.71–1.62; I²=31%; *P*=0.75). However, the differences found in the mortality outcomes were non-significant (Figs. 2A-D, 3A-C).

### Cross-clamp, CPB, and operative times

**Figure 4:**
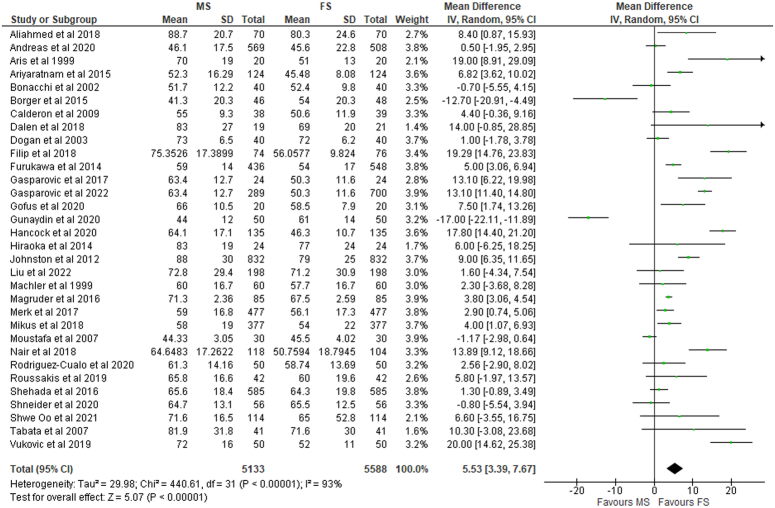
Cross clamp time in patients undergoing mini-sternotomy versus full sternotomy. FS, full sternotomy; IV, inverse variance; MS, mini-sternotomy.

**Figure 5:**
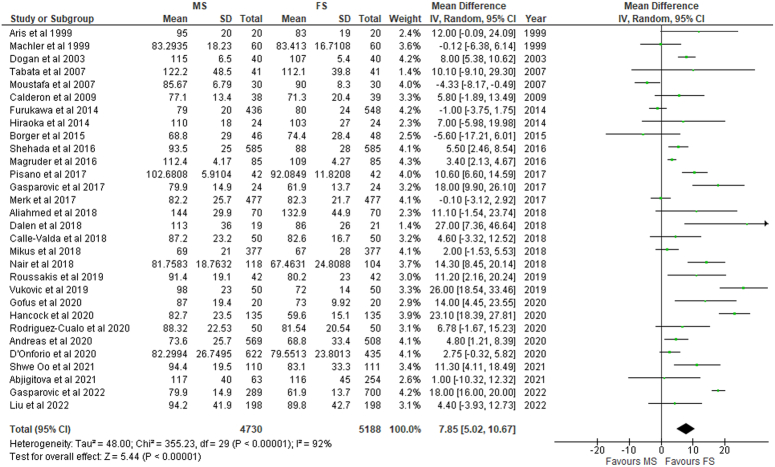
Cardiopulmonary bypass time in patients undergoing mini-sternotomy versus full sternotomy. FS, full sternotomy; IV, inverse variance; MS, mini-sternotomy.

Cross-clamp duration was significantly longer for MS (MD: 5.53; 95% CI: 3.39–7.67; I²=92%; *P*<0.01) (Fig. 4) but insignificant for the RMT group (MD: 2.26; 95% CI: −5.32 to 9.84; I²=94%; *P*=0.56) (SDC 2, Fig S20, Supplemental Digital Content 2, http://links.lww.com/MS9/A499) compared to conventional FS. Significantly greater CPB time (MD: 7.85; 95% CI: 5.02–10.67; I²=92%; *P*<0.01) (Fig. 5) was also observed; however, the operative time differences between the MS and FS group were non-significant (MD: 4.43; 95% CI: −2.43 to 11.28; I²=87%; *P*=0.21) (SDC 2, Fig S21, Supplemental Digital Content 2, http://links.lww.com/MS9/A499). This was in contrast to patients undergoing RMT, where non-significant differences were found for both CBP (*P*=0.56) and operative (*P*=0.63) time when compared with FS (SDC 2, Fig S22, Supplemental Digital Content 2, http://links.lww.com/MS9/A499).

### ICU and hospital length of stay (days)

**Figure 6:**
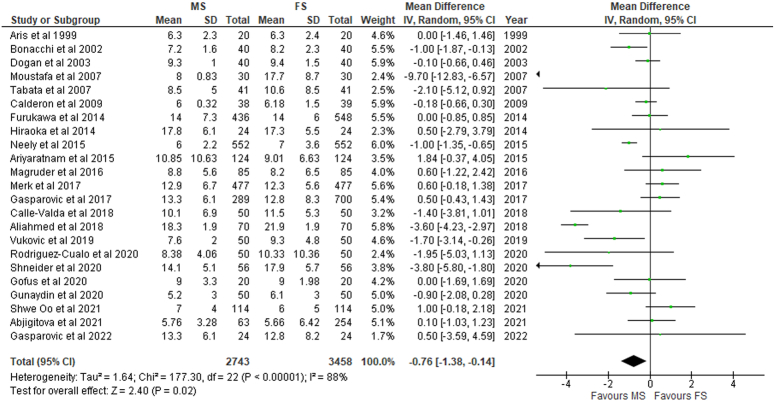
Length of stay (days) in the hospital in patients undergoing mini-sternotomy versus full sternotomy. FS, full sternotomy; IV, inverse variance; MS, mini-sternotomy.

**Figure 7:**
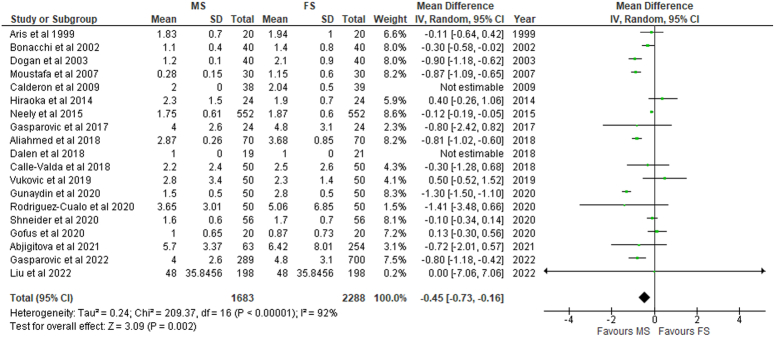
ICU in patients undergoing mini-sternotomy versus full sternotomy. FS, full sternotomy; IV, inverse variance; MS, mini-sternotomy.

Patients undergoing MS required significantly fewer days in the ICU (MD: −0.45; 95% CI: −0.73 to −0.16; I²=92%; *P*<0.01) and the hospital (MD: −0.76; 95% CI: −1.38 to −0.14; I²=88%; *P*=0.02) (Figs. 6–7). Whereas patients in the RMT group spent significantly fewer days in the hospital (RR: −1.45; 95% CI: −2.48 to −0.42; I²=76%; *P*<0.01) only (SDC 2, Fig S23, Supplemental Digital Content 2, http://links.lww.com/MS9/A499).

Leave-one-out sensitivity analysis showed Hiroaka *et al.* ^15^. to have a disproportionate effect on the heterogeneity of the RMT group for hospital duration results. Removal of this study led to a reduction in heterogeneity while the results remained significant (MD: −1.02; 95% CI: −1.33 to −0.72; I²=0%; *P*<0.01) (SDC 2, Fig S23, Supplemental Digital Content 2, http://links.lww.com/MS9/A499).

### Cardiac and neurological events

Complete AV block (RR: 0.55; 95% CI: 0.32–0.94; I²=0%; *P*=0.03) was significantly lower for patients undergoing MS. However, non-significant differences were observed between MS and FS for stroke, TIA, low cardiac output, permanent pacemaker, postoperative myocardial infarction (MI), atrial fibrillation, arrhythmia and intra-aortic balloon pump (IABP) insertion (SDC 2, Fig 24, Supplemental Digital Content 2, http://links.lww.com/MS9/A499). Similar results were obtained in the RMT intervention, where all cardiac outcomes reported non-significant differences (SDC 2, Fig S25, Supplemental Digital Content 2, http://links.lww.com/MS9/A499).

### Hematological events

The mean amount of blood drained (ml) in 24 h was significantly lower in patients undergoing MS when compared to FS (MD: −128.55B; 95% CI: −178.20 to −78.9; I²=93%; *P*<0.01). Patients that underwent MS presented with mild (+2) and moderate (+3) paravalvular leak complications more frequently compared to those opting for conventional FS. However, these differences were non-significant (*P*=0.38 and *P*=0.52, respectively) (SDC 2, Fig S26, Supplemental Digital Content 2, http://links.lww.com/MS9/A499). Non-significant differences were also observed between MS and FS for major bleeding, red cell transfusion (>3), reoperation for bleeding and PCV transfusion (SDC 2, Fig S26, Supplemental Digital Content 2, http://links.lww.com/MS9/A499). The latter two outcomes also reported non-significant differences between the RMT and FS group (SDC 2, Fig S27, Supplemental Digital Content 2, http://links.lww.com/MS9/A499).

### Pulmonary events

Respiratory insufficiency occurred less frequently in the MS group when compared with FS (RR: 0.64; 95% CI: 0.51–0.81; I²=0%; *P*<0.01). However, non-significant differences were observed for complications like pneumonia (*P*=0.61) and respiratory failure (*P*=0.88) (SDC 2, Fig S28, Supplemental Digital Content 2, http://links.lww.com/MS9/A499). Non-significant results were obtained for respiratory insufficiency and pneumonia in the RMT versus FS group (SDC 2, Fig S28, Supplemental Digital Content 2, http://links.lww.com/MS9/A499).

### Renal events

Renal insufficiency occurred significantly less frequently in patients undergoing MS (RR: 0.54; 95% CI: 0.40–0.72; I²=0%; *P*<0.01) whereas non-significant results were obtained for those in the RMT group (RR: 0.62; 95% CI: 0.30–1.29; I²=0%; *P*=0.20) (SDC 2, Fig S29, Supplemental Digital Content 2, http://links.lww.com/MS9/A499). New onset dialysis occurred less frequently in both the minimally invasive groups; however, results obtained were non-significant. Moreover, the renal failure outcome also presented with non-significant findings in the MS versus FS group (*P*=0.980) (SDC 2, Fig S29, Supplemental Digital Content 2, http://links.lww.com/MS9/A499).

### Ventilation time (h) and extracorporeal membrane oxygenation (ECMO)

**Figure 8:**
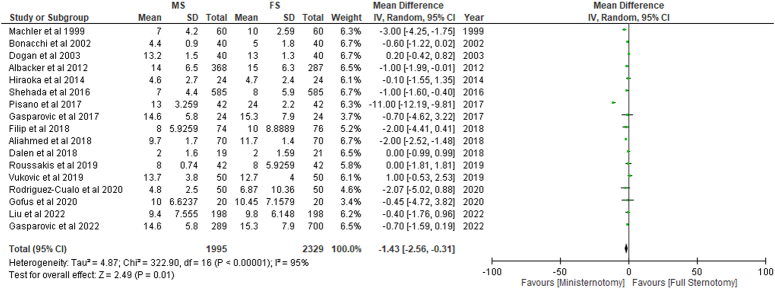
Ventilation per 24 h in patients undergoing mini-sternotomy versus full sternotomy. FS, full sternotomy; IV, inverse variance; MS, mini-sternotomy.

Significantly lower time for ventilation (h) was required for patients undergoing MS (MD: −1.43; 95% CI: −2.56 to −0.31; I²=95%; *P*=0.01) (Fig. 8) and RMT (MD: −1.81; 95% CI: −2.70 to −0.92; I²=0%; *P*<0.01) when compared with conventional treatment. ECMO was required less frequently in patients undergoing MS; however, the results obtained were non-significant (*P*=0.39) (SDC 2, Fig S30, Supplemental Digital Content 2, http://links.lww.com/MS9/A499).

### Surgical site infections and sternum re-fixation

Sternal wound infection was the most common surgical site infection; however, differences were non-significant for both the MS group (*P*=0.96) and RMT group (*P*=0.17) when compared with the conventional FS treatment (SDC 2, Fig S31, Supplemental Digital Content 2, http://links.lww.com/MS9/A499). Results obtained for sternum re-fixation in the MS versus FS group were also non-significant (*P*=0.68) (SDC 2, Fig S31, Supplemental Digital Content 2, http://links.lww.com/MS9/A499).

## Discussion

We conducted a meta-analysis of 48 studies, including 15 RCTs and 33 observational studies, to compare the results of patients who underwent surgical AVR with minimally invasive approaches, including RMT and FS. In a recent meta-analysis by Chang *et al.* ^16^, the three methods for AVR were examined using PSM studies. Another recent meta-analysis, which included observational studies evaluated the effects of minimally invasive techniques, RMT, and MS for AVR^17^. We included both RCTs and PSM studies in our analysis.

In line with the meta-analysis conducted by Ogami *et al.* ^8^. in which patients who received AVR with MS compared to FS had considerably decreased operative mortality, our study found that outcomes, including 30-day, operative, and inpatient mortality occurred less frequently in MS than in patients with FS, although 1-year mortality occurred more often. However, the variations in mortality outcomes were not significant statistically (RR: 1.07; 95% CI: 0.71–1.62; I²=31%; *P*=0.75). The mortality outcomes in patients undergoing RMT versus FS were also non-significant. While deciding on a surgical strategy, physicians will find solace in the current study’s comparable mortality rates. Although a minimally invasive technique during AVR may alter immediate results, other factors such as age, atrial fibrillation, left ventricular dysfunction, along with the existence of cardiovascular illness may have an impact on long-term survival, as indicated in the literature^18,19^.

The current study demonstrated significantly shorter hospital stays (MD: −0.76; 95% CI: −1.38 to −0.14; I²=88%; *P*=0.02) and ICU stay (MD: −0.45; 95% CI: −0.73 to −0.16; I²=92%; *P*<0.01) with MS, which is consistent with prior studies^5,20^. This might be due to the conservation of sternal stability, which can lead to better pulmonary status and fewer sternal issues^21^. The decline in length of stay in the MIAVR group may be a predictor of decreased postoperative complications. Similarly, patients in the RMT group had significantly shorter hospital stay (RR: −1.45; 95% CI: −2.48 to −0.42; I^2^=76%; *P* 0.01).

Compared to the conventional FS, patients who received minimally invasive approaches, including MS (MD: −1.43; 95% CI: −2.56 to −0.31; I^2^=95%; *P*=0.01) and RMT (MD: −1.81; 95% CI: −2.70 to −0.92; I^2^=0%; p0.01) required significantly less time for ventilation (hours). Although patients who received MS required extracorporeal membrane oxygenation (ECMO) less frequently, this difference was not statistically significant (*P*=0.39).

Minimally invasive approaches such as RMT may have an advantage over MS in reducing the risk of infection and wound complications, since the sternum and rib bones are not disrupted during the procedure^5^. However, our meta-analysis did not find evidence to support this potential benefit. Although sternal wound infection was the most frequent surgical site infection, the differences in infection rates were not statistically significant for either the MS group (*P*=0.96) or the RMT group (*P*=0.17) compared to the standard FS treatment. Additionally, there were no statistically significant differences in sternum re-fixation rates between the MS and FS groups (*P*=0.68).

AV block in its entirety was found to be considerably lower for participants receiving MS treatment (RR: 0.55; 95% CI: 0.32–0.94; I²=0%; *P*=0.03). No significant differences were observed between MS and FS for poor cardiac output, permanent pacemaker, postoperative MI, atrial fibrillation, arrhythmia, or IABP. Similar outcomes were also observed with the RMT intervention, with no significant differences in any of the cardiac outcomes. While previous concerns have been raised about the typical RMT approach requiring retrograde arterial perfusion via the femoral artery, which has been associated with an increased risk of stroke^22^, our analysis did not identify any significant differences in this area.

Patients undergoing MS had much less blood drained on average (ml) in a 24-h period than those undergoing FS (MD: −128.55B; 95% CI: −178.20 to −78.9; I²=93%; *P*<0.01). This is also reinforced by Brown *et al.* ^5^. Minimal surgical trauma may be linked to lessened blood loss. Significant differences in serious bleeding, red cell transfusion (>3), reoperation for bleeding, and PCV transfusion were not found between MS and FS. The latter two results also showed no statistically significant differences between the FS and RMT groups.

When compared to FS, the MS group experienced respiratory (*P*=0.01) and renal insufficiencies (*P*=0.01) less frequently. Intuitively, these benefits may be especially relevant for elderly patients who are at a higher risk, have lower physiological resources, and may better withstand minimally invasive procedures, recovering more quickly^23^.

Even though prior studies including a Bayesian network meta-analysis by K Phan *et al.* ^24^. have found RMT to have increased CPB time and cross-clamp duration, no significant differences were found to exist in cross-clamp duration (*P*=0.56) and CPB time (*P*=0.56) between RMT and FS in our study. This contrasts with patients undergoing MS, where cross-clamp duration (MD: 5.53; 95% CI: 3.39–7.67; I²=92%; *P*<0.01) and CPB time (MD: 7.85; 95% CI: 5.02–10.67; I²=92%; *P*<0.01) was significantly longer for MS as compared to FS.

It must be noted that studies did not always report similar outcomes, because there are no established criteria for assessing the clinical utility of less-invasive cardiac surgical procedures. Important measures such as quality of life and return to work were only reported in a handful of studies. Significant heterogeneity was detected in the operative parameters including CPB and cross-clamp duration, which may be due to different patient cohort characteristics and selection criteria among the studies. The heterogeneity present in results may also reflect the variation in experience and expertise of different centers, given that MIAVR is associated with a significant learning curve. To achieve the best clinical results, minimally invasive techniques are more technically complex than FS and call for more specialized training. Future minimally invasive procedures may be made easier by the growing usage of suture less implantation techniques, which could lead to further shorter CPB and cross-clamp durations^25^.

Cardiothoracic surgeons have certain concerns about the CPB method used during MIAVR. These concerns involve restricted cardiac exposure, which poses various theoretical dangers such as cannula insertion and appropriate cardioplegia. Minimal exposure could prevent the placement of pacing wires or the insertion of a retrograde cardioplegia cannula. Some of these issues are mitigated by transesophageal echocardiography, as well as by injecting CO2 into the surgical area and manipulating vacant ventricles on CPB to prevent right ventricular damage^26^.

There has been little research done on the cost of mini-AVR, though it has been criticized for being more costly due to longer operating hours and the need for specialized equipment. In comparison to patients who underwent conventional AVR, the sum of hospital expenses was 5% less for patients who underwent mini-AVR, according to a multi-institutional analysis conducted by Ghanta *et al.* ^27^. More frequent early discharges and less usage of blood products were the main causes of lower total expenses.

### Limitations

Minimally invasive surgery is becoming more popular among younger people and is being performed by highly skilled surgeons. Furthermore, minimally invasive surgery is not used on patients who are at high risk. Patients with extensive atherosclerosis, impaired respiratory function, or advanced age are not candidates for the minimally invasive method. For these reasons, the conditions of the patients and the surgeon’s skills should be examined in future research projects.

## Conclusion

While mortality rates are comparable in minimally invasive approaches and FS, MS appears to be a safer approach than both RMT and FS. This is likely due fewer respiratory and renal insufficiencies, as well as shorter hospital and ICU stay. Overall, the use of minimally invasive approaches for AVR may provide significant benefits in terms of reduced hospital stay and postoperative complications, making it a viable option for patients. However, further studies are needed to investigate the long-term outcomes and potential complications of these approaches.

## Ethical approval

Not applicable.

## Consent

Not applicable.

## Source of funding

Not applicable.

## Author contribution

S.K. made substantial contributions to the conception or design of the study and drafted the manuscript. A.A. contributed in analyses of the data. M.H. and M.S.S. participated in data collection, literature search, and revised the manuscript. F.A. and S.S. supervised and revised the article critically for intellectual content. All authors read and approved the final manuscript.

## Conflicts of interest disclosure

The authors declare no conflicts of interest.

## Research registration unique identifying number (UIN)

We registered this protocol with Prospero (CRD42023453991).

## Guarantor

Saad Khalid.

## Data availability statement

Data are available upon request from the corresponding author.

## Provenance and peer review

Not commissioned, externally peer-reviewed.

## Supplementary Material

SUPPLEMENTARY MATERIAL
