# Supplementary material for: Minimally invasive approaches versus conventional sternotomy for aortic valve replacement in patients with aortic valve disease: a systematic review and meta-analysis of 17 269 patients
Source: Ann Med Surg (Lond). 2024 Jun 4;86(7):4005–14. doi: 10.1097/MS9.0000000000002204 (PMC11230795; doi:10.1097/MS9.0000000000002204)
Supplement: SUPPLEMENTARY MATERIAL [file ms9-86-4005-s001.docx]

| **Author** | **Year** | **Country** | **Period** | **Design RCT/PSM** | **Number of Patients** | | **Type of minimally  invasive surgery MS/RMT** | **Conversion  rate (%)** | **Mean or median CPB time (min)** | | **Mean or median followup (year)** | **Age (Mean or median)** | | **Female (%)** | | **Type of operative  risk score Euro/Euro II/ LE/ STS** | **Operative risk score (Mean or median)** | |
| --- | --- | --- | --- | --- | --- | --- | --- | --- | --- | --- | --- | --- | --- | --- | --- | --- | --- | --- |
|  |  |  |  |  | **Minimally invasive** | **FS** |  |  | **Minimally invasive** | **FS** |  | **Minimally invasive** | **FS** | **Minimally invasive** | **FS** |  | **Minimally invasive** | **FS** |
| Machler [1] | 1999 | Austria | 1996-1997 | RCT | 20 | 20 | MS | N/A | 115 | 107 | 0.8 | 65 [56-70] | 65 [55-72] | 41.7 | 40 | N/A | N/A | N/A |
| Aris [2] | 1999 | Spain | N/A | RCT | 20 | 20 | MS | 10 | 95 | 83 | N/A | 66.5 ± 10 | 62.2 ±12 | N/A | N/A | N/A | 11.6 ± 6 5 | 11.4 ± 5.5 |
| Bonacchi [3] | 2002 | Italy | 1999-2001 | RCT | 40 | 40 | MS | 2.5 | N/A | N/A | 0.8 | 62.6 ± 9.5 | 64 ± 12.4 | N/A | N/A | N/A | N/A | N/A |
| Dogan [4] | 2003 | Germany | N/A | RCT | 40 | 40 | MS | N/A | 107 | 115 | 0.16 | 65.7 ± 1.9 | 64.3 ± 2.9 | 22 | 18 | - | - | - |
| Moustafa [5] | 2007 | Egypt | N/A | RCT | 30 | 30 | MS | 6.7 | 85.7 | 90 | N/A | 22.9 ± 2.4 | 23.8 ± 3.5 | 46.7 | 50 | N/A | N/A | N/A |
| Tabata [6] | 2007 | USA | 1996-2005 | PSM | 41 | 41 | MS | 0 | 122 | 112.1 | N/A | 65.8 ± 13.6 | 67.6 ± 15.2 | 14.6 | 24.4 | N/A | N/A | N/A |
| Calderon [7] | 2009 | France | 2003-2007 | RCT | 38 | 39 | MS | N/A | 77.1 | 71.3 | N/A | 70.9 ± 11.4 | 70.8 ± 10.2 | 39.5 | 30.8 | Euro | 5.4 ± 1.9 | 5.2 ± 1.8 |
| Ruttmann [8] | 2010 | Austria | 2006-2009 | PSM | 87 | 87 | RMT | 1.1 | 151.4 | 129 | 1.5 | 70 ± 9.2 | 67 ± 13.4 | 46 | 36 | Euro | 5.71 ± 2.16 | 5.74 ± 2.23 |
| Murzi [9] | 2012 | Italy | 2006-2011 | PSM | 100 | 100 | RMT | N/A | 119 | 106 | 3.5 | 65.4 ± 9 | 64.6 ± 9 | 70 | 72 | Euro | 4.6 [2–14] | 4.6 [2–18] |
| Ahangar [10] | 2013 | India | 2010-2012 | PSM | 30 | 30 | RMT | N/A | 122.1 | 121.8 | N/A | 38.5 ± 10.6 | 36.6 ± 6.7 | 73.3 | 56.6 | N/A | N/A | N/A |
| [Johnston](https://pubmed.ncbi.nlm.nih.gov/?term=Johnston+DR&cauthor_id=22244556) [11] | 2012 | USA | 1995 -2004 | PSM | 832 | 832 | MS | 2.85 | N/A | N/A | JS 6.8 /FS 6.3 | 59 ± 16 | 61 ± 16 | 33 | 36 | STS | N/A | N/A |
| Glauber [12] | 2013 | Italy | 2005-2010 | PSM | 138 | 138 | RMT | 1.5 | N/A | N/A | 2.5 | 69.5 ± 12.4 | 69.8 ± 11.6 | 42 | 39.1 | Euro | 5.6 [3-8.1] | 5.4 [3-7.5] |
| Hiraoka [13] | 2014 | Japan | 2007-2012 | PSM | 24 (MS)/ 36 (RMT) | 24/36 | MS/RMT | N/A | 110 (MS)/ 129 (RMT) | 103/125 | N/A | 74.6 ± 9.5/ 62.6 ± 10.7 | 70.8 ± 13.2/ 62.5 ± 14.0 | 50/56 | 33/53 | STS | 110/ 1.08 ± 0.84 | 103/ 1.30 ± 0.98 |
| Furukawa [14] | 2014 | Japan | 2009-2012 | PSM | 404 | 404 | MS | N/A | 59 | 54 | N/A | 69 ± 12 | 69 ± 11 | 44 | 46 | Euro | 6 ± 5 | 6 ± 4 |
| Albacker [15] | 2014 | USA | 1995-2010 | PSM | 223 | 223 | MS | N/A | N/A | N/A | 2.7 | 67 ± 13 | 68 ± 13 | 47 | 47 | N/A | N/A | N/A |
| Neely [16] | 2015 | USA | 2002-2015 | PSM | 552 | 552 | MS | N/A | 106 | 124 | N/A | 74.5 ± 10.7 | 74.6 ± 10.7 | 29.3 | 23.3 | N/A | N/A | N/A |
| Gilmanov [17] | 2015 | Italy | 2001-2013 | PSM | 100 | 100 | RMT | 2 | 92 | 94 | 2.8 | 82.5 ± 2.2 | 83.0 ± 2.1 | 67 | 64 | EI/ LE | 8 [8–9]/ 10.2 [8.4–14.6] | 8 [8–9]/ 10.5 [8.4–14.6] |
| Merk [18] | 2015 | Europe | 2003-2012 | PSM | 477 | 477 | MS | 0.8 | 82.2 | 81 | 3.1 | 67.8 ± 11.2 | 67.5 ± 11.0 | 41.5 | 44 | LE | 6.6 ± 6.4 | 6.6 ± 6.8 |
| Ariyaratnam [19] | 2015 | UK | 1999-2013 | PSM | 124 | 124 | MS | 0 | 68.9 | 62.7 | 0.5 | 65.5 ± 14.3 | 65.4 ± 14.3 | 52.4 | 51.6 | LE | 6.48 ± 6.6 | 5.52 ± 7.1 |
| Borger [20] | 2015 | Germany | 2012-2013 | RCT | 46 | 48 | MS | N/A | 68.8 | 74.4 | N/A | 73.0 ± 5.3 | 74.2 ± 5.0 | 19 | 27 | STS | 1.6 ± 0.7 | 1.7 ± 0.6 |
| Borger [21] | 2016 | USA/Germany | N/A | RCT | 46 | 48 | MS | N/A | 68.8 | 74.4 | 1 | 73.0 ± 5.3 | 74.2 ± 5.0 | 41 | 56 | LE/Euro II/ STS | 6.4 ± 3.7/ 1.7 ± 0.9/1.6 ± 0.7 | 6.7 ± 3.6/1.8 ± 1.0/1.7 ± 0. |
| Magruder [22] | 2016 | USA | 2008-2013 | PSM | 85 | 85 | MS | N/A | 112.4 | 109 | N/A | 65.5 ± 13.4 | 65.1 ± 14.4 | 38.8 | 30.6 | N/A | N/A | N/A |
| Shehada [23] | 2016 | Germany | 2001-2012 | PSM | 585 | 585 | MS | N/A | 93.5 | 88 | N/A | 65.0 ± 10.5 | 65.7 ± 11.5 | 37.2 | 37.2 | N/A | N/A | N/A |
| Semsroth [24] | 2017 | Europe | 2005-2013 | PSM | 118 (MS)/ 118 (RMT) | 118 | MS/RMT | 4.4/12 | 114 (MS)/ 141 (RMT) | 110 | 1 | 72 [38–91]/ 72 [34–89] | 73 [41–93] | 49.2/ 45.8 | 49.2 | N/A | N/A | N/A |
| Gasparovic [25] | 2017 | Slovakia | 2010-2013 | PSM | 24 | 24 | MS | 0 | 79.9 | 61.9 | N/A | 60.2 ± 15.8 | 65 ± 7.2 | 50 | 50 | Euro II | 1.22 ± 0.63 | 1.36 ± 1.11 |
| Pisano [26] | 2017 | Italy | N/A | PSM | 42 | 42 | MS | N/A | 103 | 94 | N/A | 76 [59–79] | 76.5 [48–85] | 64 | 50 | LE | 5.0 [4.4–7.2] | 6.4 [4.9–7.2] |
| Filip [27] | 2018 | Poland | N/A | PSM | 74 | 76 | MS | N/A | 127 | 97 | N/A | 68 (57–77) | 66 (52–73) | 47.3 | 38.2 | Euro | 7 (3–10) | 7 (3–11) |
| Nair [28] | 2018 | UK | 2010-2015 | RCT | 118 | 104 | MS | 5 | 80 | 66 | 2 | 71.3 ± 12.3 | 72.1 ± 10.9 | 45 | 55 | E | 5.9 ± 2.1 | 6.1 ± 2.1 |
| Mikus [29] | 2018 | Italy | 2010-2017 | PSM | 377 (MS)/ 377 (RMT) | 377 | MS/RMT | N/A | 69 (MS)/ 57 (RMT) | 67 | N/A | 74.1 ± 9.2/ 74.2 ± 9.9 | 73.2 ± 9.3 | 50.1/ 50.9 | 50.9 | LE | 7.9 ± 6.2/ 6.3 ± 4.3 | 11.4 ± 12.4 |
| Calle-Valda [30] | 2018 | Spain | 2011-2015 | PSM | 50 | 50 | MS | 2 | 87.2 | 82.6 | 4 | 82.3 ± 4.8 | 84.2 ± 5.1 | 44 | 64 | Euro | 8.3 ± 3.4 | 11.4 ± 3.6 |
| Aliahmed [31] | 2018 | Lithuania | 2011-2016 | PSM | 70 | 70 | MS | N/A | 144 | 132.9 | N/A | 60.8 ± 11.6 | 61.4 ± 11.9 | 40 | 40 | N/A | N/A | N/A |
| Dalen [32] | 2018 | Sweden | 2014-2015 | RCT | 19 | 21 | MS | 5 | 113 | 86 | N/A | 67 ± 9.0 | 70 ± 7.9 | 37 | 38 | Euro II | 1.26 ± 0.65 | 1.44 ± 0.90 |
| Roussakis [33] | 2019 | Greece | 2016-2019 | PSM | 42 | 42 | MS | N/A | 91.4 | 80.2 | N/A | 68.9 ± 11.0 | 68.6 ± 11.6 | 33.3 | 33.3 | Euro II | 1.45 ± 0.93 | 1.45 ± 0.89 |
| Seitz [34] | 2019 | Australia | 2013-2016 | PSM | 53 | 53 | RMT | 5.66 | 112 | 98 | N/A | 73.0 ± 8.8 | 72.0 ± 11.6 | 49.1 | 52.8 | N/A | N/A | N/A |
| Vukovic [35] | 2019 | Serbia | 2016-2017 | RCT | 50 | 50 | MS | 4 | 98 | 72 | 2 | 65 ± 8.9 | 67.8 ± 8.7 | 56 | 44 | Euro II | 1.87 ± 1.03 | 1.98 ± 1.8 |
| Gunaydin [36] | 2020 | Turkey | 2017-2019 | PSM | 50 | 50 | MS | N/A | N/A | N/A | N/A | 68 ± 10 | 67 ± 10 | 33 | 39 | STS | 9.4 ± 1 | 10.5 ± 1 |
| Shneider [37] | 2020 | Russia | 2012-2017 | RCT | 56 | 56 | MS | 5.3 | N/A | N/A | 2.7 | 53.1±14.9 | 56.1±14.3 | 57.1 | 55.4 | Euro II | 2.3±0.7 | 2.6±0.5 |
| Andreas [38] | 2020 | International | 2015-2018 | PSM | 569 (MS)/ 569 (RMT) | N/A | MS/RMT | 0.9 (MS)/ 1 (RMT) | 73.6 (MS)/ 90.3 (RMT) | N/A | N/A | 77.4 ± 6.7/ 75.8 ± 6.8 | 77.5 ± 7.3 | 64.4/ 62.5 | 65.9 | LE | 10.7 ± 8.5/ 8.7 ± 6.3 | 11 ± 8.6 |
| Hancock [39] | 2021 | UK | 2014-2016 | RCT | 135 | 135 | MS | 11.8 | 82.7 | 59.6 | N/A | 69.3 ± 9.3 | 68.7 ± 8.4 | 42.2 | 35.6 | LE/ Euro II | 5.2 ± 3.5/ 1.5 ± 1.1 | 5.1 ± 3.5/ 1.5 ± 1.2 |
| Oo [40] | 2021 | UK | 2015-2020 | PSM | 114 | 114 | MS | N/A | 94.4 | 83.1 | 3.4 | 71.3 ± 9.4 | 68.5 ±11.5 | 44.5 | 37.4 | LE | 5.7 ± 3.7 | 5.8 ± 5.1 |
| Meyer [41] | 2021 | Germany | 2015-2019 | PSM | 85 | 85 | RMT | 0 | 105 | 87 | 1.5 | 67.1 ± 11.9 | 66.8 ± 10.4 | 41 | 39 | Euro II/ STS | 1.30 ± 0.88/ 1.46 ± 0.99 | 1.28 ± 0.73/ 1.30 ± 1.16 |
| Bonacchi [42] | 2021 | International | 1999-2019 | PSM | 986 (MS)/ 986 (RMT) | N/A | MS/RMT | 9 (MS)/ 23 (RMT) | 56.7 (MS)/ 65.5 (RMT) | N/A | 10 | 71.2 ± 9 | 71.5 ± 10 | 60 | 60 | Euro II/ STS | 6.1 ± 3.2/ 0.28 ± 0.15 | 6.0 ± 3.4/ 0.31 ± 22 |
| Rodriguez-Caulo [43] | 2021 | Spain | 2016-2018 | RCT | 50 | 50 | MS | 6 | 88.3 | 81.5 | N/A | 66.2 ± 11.2 | 67.6 ± 7.5 | 46 | 40 | LE | 5.2 ± 4.2 | 4.3 ± 2.1 |
| Gofus [44] | 2021 | Czech Republic | 2017-2019 | RCT | 20 | 20 | MS | 10 | 87 | 73 | N/A | N/A | N/A | 45 | 20 | Euro II | 1.5 [1-1.9] | 1.1 [0.8-1.6] |
| D'Onofrio [45] | 2021 | Italy | 2012-2019 | PSM | 622 | 435 | MS | 2.5 | 83 | 78.5 | 1 | 75.0 [69.3-79.0] | 75.0 [69.7-79.0] | 52.7 | 50.8 | STS | 1.79 [1.23-2.52] | 1.81 [1.26-2.70] |
| Abjigitova [46] | 2021 | Netherlands | N/A | PSM | 63 | 254 | MS | 15.9 | 118 | 110 | 2.9 | 68.5 [61.9-75.6] | 68.2 [59.1-73.6] | 33.3 | 39.8 | Euro II | 1.50 ±1.16 | 1.44 ±1.00 |
| Liu [47] | 2022 | China | 2014- 2021 | PSM | 198 | 198 | MS | N/A | 89.8 | 94.2 | N/A | 54.9 ± 12.7 | 53.2 ± 13.8 | 71 | 60 | Euro, Sinoscore | 2 [0–3] | 2 [0–3] |
| Gasparovic [48] | 2022 | Croatia | 2010- 2020 | PSM | 289 | 799 | MS | N/A | 79.9 | 61.9 | 5 | 60.2±15.8 | 65±7.2 | 12 | 12 | Euro II | 1.22±0.63 | 1.36±1.11 |

**Table S1:** Baseline characteristics of included studies and participants

[Euro, EuroScore; Euro II, EuroSCORE II; FS, full sternotomy; LE, logistic EuroSCORE; MS, mini-sternotomy; N/A, non-available; PSM, propensity score matching; RCT, randomized controlled trial; RMT, right mini-thoracotomy; STS, STS score]

| **Study** | **Selection** | **Comparability** | **Outcome** | **Total** |
| --- | --- | --- | --- | --- |
| Tabata [6] | 4 | 2 | 2 | 8 |
| Ruttmann [8] | 4 | 2 | 3 | 9 |
| Murzi [9] | 4 | 2 | 3 | 9 |
| Johnston [11] | 4 | 2 | 3 | 9 |
| Glauber [12] | 4 | 2 | 2 | 8 |
| Hiraoka [13] | 3 | 2 | 1 | 6 |
| Furukawa [14] | 4 | 2 | 1 | 7 |
| Albacker [15] | 4 | 2 | 3 | 9 |
| Neely [16] | 4 | 2 | 1 | 7 |
| Gilmanov [17] | 4 | 2 | 3 | 9 |
| Merk [18] | 4 | 2 | 3 | 9 |
| Ariyaratnam [19] | 4 | 2 | 3 | 9 |
| Magruder [22] | 4 | 2 | 2 | 8 |
| Shehada [23] | 4 | 2 | 2 | 8 |
| Semsroth [24] | 4 | 2 | 1 | 7 |
| Gasparovic [25] | 3 | 2 | 2 | 7 |
| Pisano [26] | 4 | 2 | 1 | 7 |
| Filip [27] | 4 | 2 | 2 | 8 |
| Mikus [29] | 4 | 2 | 1 | 7 |
| Calle-Valda [30] | 4 | 2 | 1 | 7 |
| Aliahmed [31] | 3 | 2 | 1 | 6 |
| Dalen [32] | 4 | 2 | 2 | 8 |
| Roussakis [33] | 3 | 2 | 1 | 6 |
| Seitz [34] | 4 | 2 | 2 | 8 |
| Andreas [38] | 4 | 2 | 2 | 8 |
| Oo [40] | 4 | 2 | 1 | 7 |
| Meyer [41] | 4 | 2 | 3 | 9 |
| Bonacchi [42] | 4 | 2 | 3 | 9 |
| D'Onofrio [45] | 4 | 2 | 2 | 8 |
| Abjigitova [46] | 4 | 2 | 3 | 9 |
| Liu [47] | 4 | 2 | 2 | 8 |
| Gasparovic [48] | 3 | 2 | 2 | 7 |

**Table S2:** Newcastle-Ottawa Quality Assessment Scale for PSM Cohort Studies (A study score of 8 or higher is low risk, 6 to 7 is moderate risk, and 5 or lower is high risk of bias)

[PSM = propensity score matching]

**References:**

1. Mächler HE, Bergmann P, Anelli-Monti M, et al. Minimally invasive versus conventional aortic valve operations: a prospective study in 120 patients. The Annals of Thoracic Surgery. 1999;67(4):1001-1005. doi:10.1016/S0003-4975(99)00072-7
2. Aris A, Cámara ML, Montiel J, Delgado LJ, Galán J, Litvan H. Ministernotomy versus median sternotomy for aortic valve replacement: a prospective, randomized study. The Annals of Thoracic Surgery. 1999;67(6):1583-1587. doi:10.1016/S0003-4975(99)00362-8
3. Bonacchi M, Prifti E, Giunti G, Frati G, Sani G. Does ministernotomy improve postoperative outcome in aortic valve operation? A prospective randomized study. The Annals of Thoracic Surgery. 2002;73(2):460-465. doi:10.1016/S0003-4975(01)03402-6
4. Dogan S, Dzemali O, Wimmer-Greinecker G, et al. Minimally invasive versus conventional aortic valve replacement: a prospective randomized trial. J Heart Valve Dis. 2003;12(1):76-80.
5. Moustafa MA, Abdelsamad AA, Zakaria G, Omarah MM. Minimal vs Median Sternotomy for Aortic Valve Replacement. Asian Cardiovasc Thorac Ann. 2007;15(6):472-475. doi:10.1177/021849230701500605
6. Tabata M, Aranki SF, Fox JA, Couper GS, Cohn LH, Shekar PS. Minimally Invasive Aortic Valve Replacement in Left Ventricular Dysfunction. Asian Cardiovasc Thorac Ann. 2007;15(3):225-228. doi:10.1177/021849230701500310
7. Calderon J, Richebe P, Guibaud JP, et al. Prospective Randomized Study of Early Pulmonary Evaluation of Patients Scheduled for Aortic Valve Surgery Performed by Ministernotomy or Total Median Sternotomy. Journal of Cardiothoracic and Vascular Anesthesia. 2009;23(6):795-801. doi:10.1053/j.jvca.2009.03.011
8. Ruttmann E, Gilhofer TS, Ulmer H, et al. Propensity score-matched analysis of aortic valve replacement by mini-thoracotomy. J Heart Valve Dis. 2010;19(5):606-614.
9. Murzi M, Cerillo AG, Bevilacqua S, Gilmanov D, Farneti P, Glauber M. Traversing the learning curve in minimally invasive heart valve surgery: a cumulative analysis of an individual surgeon’s experience with a right minithoracotomy approach for aortic valve replacement. European Journal of Cardio-Thoracic Surgery. 2012;41(6):1242-1246. doi:10.1093/ejcts/ezr230
10. Ahangar AG, Charag AH, Wani ML, et al. Comparing Aortic Valve Replacement through Right Anterolateral Thoracotomy with Median Sternotomy. Int Cardiovasc Res J. 2013;7(3):90-94.
11. Johnston DR, Atik FA, Rajeswaran J, et al. Outcomes of less invasive J-incision approach to aortic valve surgery. J Thorac Cardiovasc Surg. 2012;144(4):852-858.e3. doi:10.1016/j.jtcvs.2011.12.008
12. Glauber M, Miceli A, Gilmanov D, et al. Right anterior minithoracotomy versus conventional aortic valve replacement: A propensity score matched study. The Journal of Thoracic and Cardiovascular Surgery. 2013;145(5):1222-1226. doi:10.1016/j.jtcvs.2012.03.064
13. Hiraoka A, Totsugawa T, Kuinose M, et al. Propensity Score-Matched Analysis of Minimally Invasive Aortic Valve Replacement. Circ J. 2014;78(12):2876-2881. doi:10.1253/circj.CJ-14-0861
14. Furukawa N, Kuss O, Aboud A, et al. Ministernotomy versus conventional sternotomy for aortic valve replacement: matched propensity score analysis of 808 patients†. European Journal of Cardio-Thoracic Surgery. 2014;46(2):221-227. doi:10.1093/ejcts/ezt616
15. Albacker TB, Blackstone EH, Williams SJ, et al. Should less-invasive aortic valve replacement be avoided in patients with pulmonary dysfunction? The Journal of Thoracic and Cardiovascular Surgery. 2014;147(1):355-361.e5. doi:10.1016/j.jtcvs.2012.12.014
16. Neely RC, Boskovski MT, Gosev I, et al. Minimally invasive aortic valve replacement versus aortic valve replacement through full sternotomy: the Brigham and Women’s Hospital experience. Ann Cardiothorac Surg. 2015;4(1):38-48. doi:10.3978/j.issn.2225-319X.2014.08.13
17. Gilmanov D, Farneti PA, Ferrarini M, et al. Full sternotomy versus right anterior minithoracotomy for isolated aortic valve replacement in octogenarians: a propensity-matched study†. Interactive CardioVascular and Thoracic Surgery. 2015;20(6):732-741. doi:10.1093/icvts/ivv030
18. Merk DR, Lehmann S, Holzhey DM, et al. Minimal invasive aortic valve replacement surgery is associated with improved survival: a propensity-matched comparison†. European Journal of Cardio-Thoracic Surgery. 2015;47(1):11-17. doi:10.1093/ejcts/ezu068
19. Ariyaratnam P, Loubani M, Griffin SC. Minimally invasive aortic valve replacement: Comparison of long-term outcomes. Asian Cardiovasc Thorac Ann. 2015;23(7):814-821. doi:10.1177/0218492315587606
20. Borger MA, Moustafine V, Conradi L, et al. A Randomized Multicenter Trial of Minimally Invasive Rapid Deployment Versus Conventional Full Sternotomy Aortic Valve Replacement. The Annals of Thoracic Surgery. 2015;99(1):17-25. doi:10.1016/j.athoracsur.2014.09.022
21. Borger MA, Dohmen PM, Knosalla C, et al. Haemodynamic benefits of rapid deployment aortic valve replacement via a minimally invasive approach: 1-year results of a prospective multicentre randomized controlled trial. Eur J Cardiothorac Surg. 2016;50(4):713-720. doi:10.1093/ejcts/ezw042
22. Magruder JT, Grimm JC, Kilic A, et al. Mini-aortic valve replacements are not associated with an increased incidence of patient–prosthesis mismatch: a propensity-scored analysis. Gen Thorac Cardiovasc Surg. 2016;64(3):144-148. doi:10.1007/s11748-015-0614-z
23. Shehada SE, Öztürk Ö, Wottke M, Lange R. Propensity score analysis of outcomes following minimal access versus conventional aortic valve replacement †. European Journal of Cardio-Thoracic Surgery. 2016;49(2):464-470. doi:10.1093/ejcts/ezv061
24. Semsroth S, Matteucci Gothe R, Raith YR, et al. Comparison of Two Minimally Invasive Techniques and Median Sternotomy in Aortic Valve Replacement. The Annals of Thoracic Surgery. 2017;104(3):877-883. doi:10.1016/j.athoracsur.2017.01.095
25. Gasparovic I, Artemiou P, Hudec V, Hulman M. Long-term outcomes following minimal invasive versus conventional aortic valve replacement: a propensity match analysis. BLL. 2017;118(08):479-484. doi:10.4149/BLL_2017_092
26. Pisano C, Totaro P, Triolo OF, Argano V. Advantages of Minimal Access versus Conventional Aortic Valve Replacement in Elderly or Severely Obese Patients. Innovations�(Phila). 2017;12(2):102-108. doi:10.1097/imi.0000000000000354
27. Filip G, Bryndza MA, Konstanty-Kalandyk J, et al. Ministernotomy or sternotomy in isolated aortic valve replacement? Early results. Kardiochir Torakochirurgia Pol. 2018;15(4):213-218. doi:10.5114/kitp.2018.80916
28. Nair SK, Sudarshan CD, Thorpe BS, et al. Mini-Stern Trial: A randomized trial comparing mini-sternotomy to full median sternotomy for aortic valve replacement. The Journal of Thoracic and Cardiovascular Surgery. 2018;156(6):2124-2132.e31. doi:10.1016/j.jtcvs.2018.05.057
29. Mikus E, Calvi S, Campo G, et al. Full Sternotomy, Hemisternotomy, and Minithoracotomy for Aortic Valve Surgery: Is There a Difference? Ann Thorac Surg. 2018;106(6):1782-1788. doi:10.1016/j.athoracsur.2018.07.019
30. Calle-Valda CM, Aguilar R, Benedicto A, et al. Outcomes of Aortic Valve Replacement According to Surgical Approach in Intermediate and Low Risk Patients: A Propensity Score Analysis. Heart, Lung and Circulation. 2018;27(7):885-892. doi:10.1016/j.hlc.2017.08.010
31. Aliahmed HMA, Karalius R, Valaika A, Grebelis A, Semėnienė P, Čypienė R. Efficacy of Aortic Valve Replacement through Full Sternotomy and Minimal Invasion (Ministernotomy). Medicina (Kaunas). 2018;54(2):26. doi:10.3390/medicina54020026
32. Dalén M, Oliveira Da Silva C, Sartipy U, et al. Comparison of right ventricular function after ministernotomy and full sternotomy aortic valve replacement: a randomized study. Interactive CardioVascular and Thoracic Surgery. 2018;26(5):790-797. doi:10.1093/icvts/ivx422
33. Roussakis A, Gavalaki A, Contrafouris C, et al. Minimally invasive aortic valve replacement: Initial experience of the 1st Cardiac Surgery Department of Onassis Cardiac Surgery Center. A propensity score-adjusted analysis. Hellenic Journal of Cardiology. 2020;61(5):346-348. doi:10.1016/j.hjc.2019.11.007
34. Seitz M, Goldblatt J, Paul E, Marcus T, Larobina M, Yap CH. Minimally Invasive Aortic Valve Replacement Via Right Anterior Mini-Thoracotomy: Propensity Matched Initial Experience. Heart, Lung and Circulation. 2019;28(2):320-326. doi:10.1016/j.hlc.2017.11.012
35. Vukovic PM, Milojevic P, Stojanovic I, et al. The role of ministernotomy in aortic valve surgery—A prospective randomized study. Journal of Cardiac Surgery. 2019;34(6):435-439. doi:10.1111/jocs.14053
36. Gunaydin S, Ozisik K, Gunertem OE, et al. Minimally Invasive Aortic Valve Replacement on Minimally Invasive Extracorporeal Circulation: Going beyond Aesthetics. J Extra Corpor Technol. 2020;52(2):90-95. doi:10.1182/ject-2000015
37. Shneider YA, Tsoi MD, Fomenko MS, Pavlov AA, Shilenko PA. Aortic valve replacement via J-shaped partial upper sternotomy: randomized trial, mid-term results. Effektivnost' i bezopasnost' protezirovaniya aortal'nogo klapana cherez «mini-J» sternotomiyu: randomizirovannoe issledovanie, sredne-otdalennye rezul'taty. Khirurgiia (Mosk). 2020;(7):25-30. doi:10.17116/hirurgia202007125
38. Andreas M, Berretta P, Solinas M, et al. Minimally invasive access type related to outcomes of sutureless and rapid deployment valves. Eur J Cardiothorac Surg. 2020;58(5):1063-1071. doi:10.1093/ejcts/ezaa154
39. Hancock HC, Maier RH, Kasim A, et al. Mini-sternotomy versus conventional sternotomy for aortic valve replacement: a randomised controlled trial. BMJ Open. 2021;11(1):e041398. doi:10.1136/bmjopen-2020-041398
40. Oo S, Khan A, Chan J, et al. Propensity matched analysis of minimally invasive versus conventional isolated aortic valve replacement. Perfusion. Published online September 13, 2021:02676591211045802. doi:10.1177/02676591211045802
41. Meyer A, van Kampen A, Kiefer P, et al. Minithoracotomy versus full sternotomy for isolated aortic valve replacement: Propensity matched data from two centers. Journal of Cardiac Surgery. 2021;36(1):97-104. doi:10.1111/jocs.15177
42. Bonacchi M, Dokollari A, Parise O, et al. Ministernotomy compared with right anterior minithoracotomy for aortic valve surgery. The Journal of Thoracic and Cardiovascular Surgery. Published online April 23, 2021. doi:10.1016/j.jtcvs.2021.03.125
43. Rodríguez-Caulo EA, Guijarro-Contreras A, Guzón A, et al. Quality of Life After Ministernotomy Versus Full Sternotomy Aortic Valve Replacement. Seminars in Thoracic and Cardiovascular Surgery. 2021;33(2):328-334. doi:10.1053/j.semtcvs.2020.07.013
44. Gofus J, Vobornik M, Koblizek V, et al. Pulmonary function and quality of life after aortic valve replacement through ministernotomy: a prospective randomized study. Kardiologia Polska (Polish Heart Journal). 2020;78(12):1278-1280. doi:10.33963/KP.15668
45. D’Onofrio A, Tessari C, Lorenzoni G, et al. Minimally Invasive vs Conventional Aortic Valve Replacement With Rapid-Deployment Bioprostheses. The Annals of Thoracic Surgery. 2021;111(6):1916-1922. doi:10.1016/j.athoracsur.2020.06.150
46. Abjigitova D, Veen KM, Mokhles MM, Bekkers JA, Oei FB, Bogers AJ. Initial clinical experience with minimally invasive surgical aortic valve replacement. J Cardiovasc Surg (Torino). 2021;62(3):268-277. doi:10.23736/S0021-9509.20.11463-0
47. Liu R, Song J, Chu J, Hu S, Wang XQ. Comparing mini-sternotomy to full median sternotomy for aortic valve replacement with propensity-matching methods. Front Surg. 2022;9:972264. Published 2022 Oct 10. doi:10.3389/fsurg.2022.972264
48. Gašparović H, Čerina P, Tokić T, et al. Propensity-score matched comparison between minimally invasive and conventional aortic valve replacement. Croat Med J. 2022;63(5):423-430. doi:10.3325/cmj.2022.63.423
