# Supplementary material for: Minimally invasive approaches versus conventional sternotomy for aortic valve replacement in patients with aortic valve disease: a systematic review and meta-analysis of 17 269 patients
Source: Ann Med Surg (Lond). 2024 Jun 4;86(7):4005–14. doi: 10.1097/MS9.0000000000002204 (PMC11230795; doi:10.1097/MS9.0000000000002204)
Supplement: SUPPLEMENTARY MATERIAL [file ms9-86-4005-s002.docx]

**Supplemental Figures**


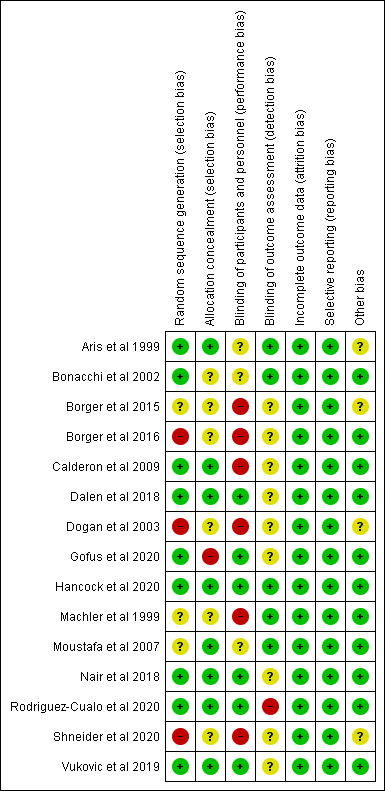


**Figure S1:** Risk assessment of Randomized Controlled trials using Cochrane risk of bias tool


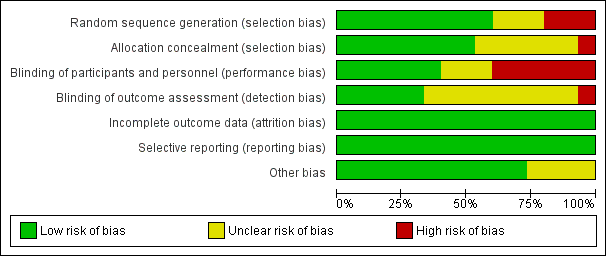


**Figure S2:** Risk of bias graph of included Randomized controlled trials using Cochrane risk of bias tool

**FUNNEL PLOTS**

**Figure S3:** Visual funnel plot representation for mortality outcomes in patients undergoing Mini-sternotomy vs Full sternotomy

1. 1 year mortality
2. 30-day mortality
3. In hospital mortality
4. Operative mortality

[SE: Standard Error; RR: Risk Ratio]


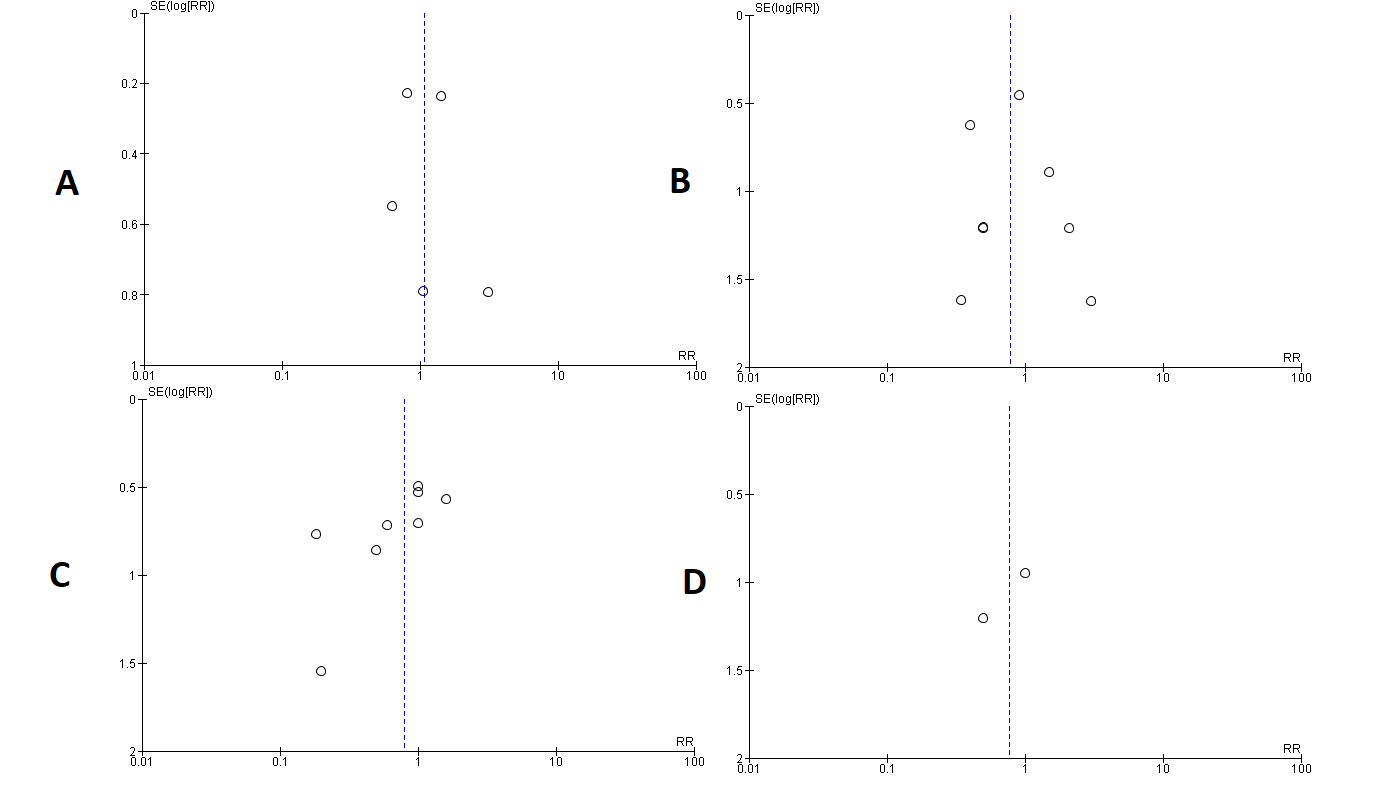


**Figure S4:** Visual funnel plot representation for cardiac outcomes in patients undergoing Mini-sternotomy vs Full Sternotomy

1. stroke

B Transient Ischemic Attack (TIA)

C - Low cardiac output

D- Complete Atrioventricular (AV) BLOCK

E- Permanent Pacemaker

F - Post operative cardiac arrhythmia

G- Post operative Atrial fibrillation (AF)

H - Post operative Intra-aortic balloon pump (IABP)

I - Post operative Myocardial Infarction (MI)

**[SE: Standard Error; RR: Risk Ratio]**


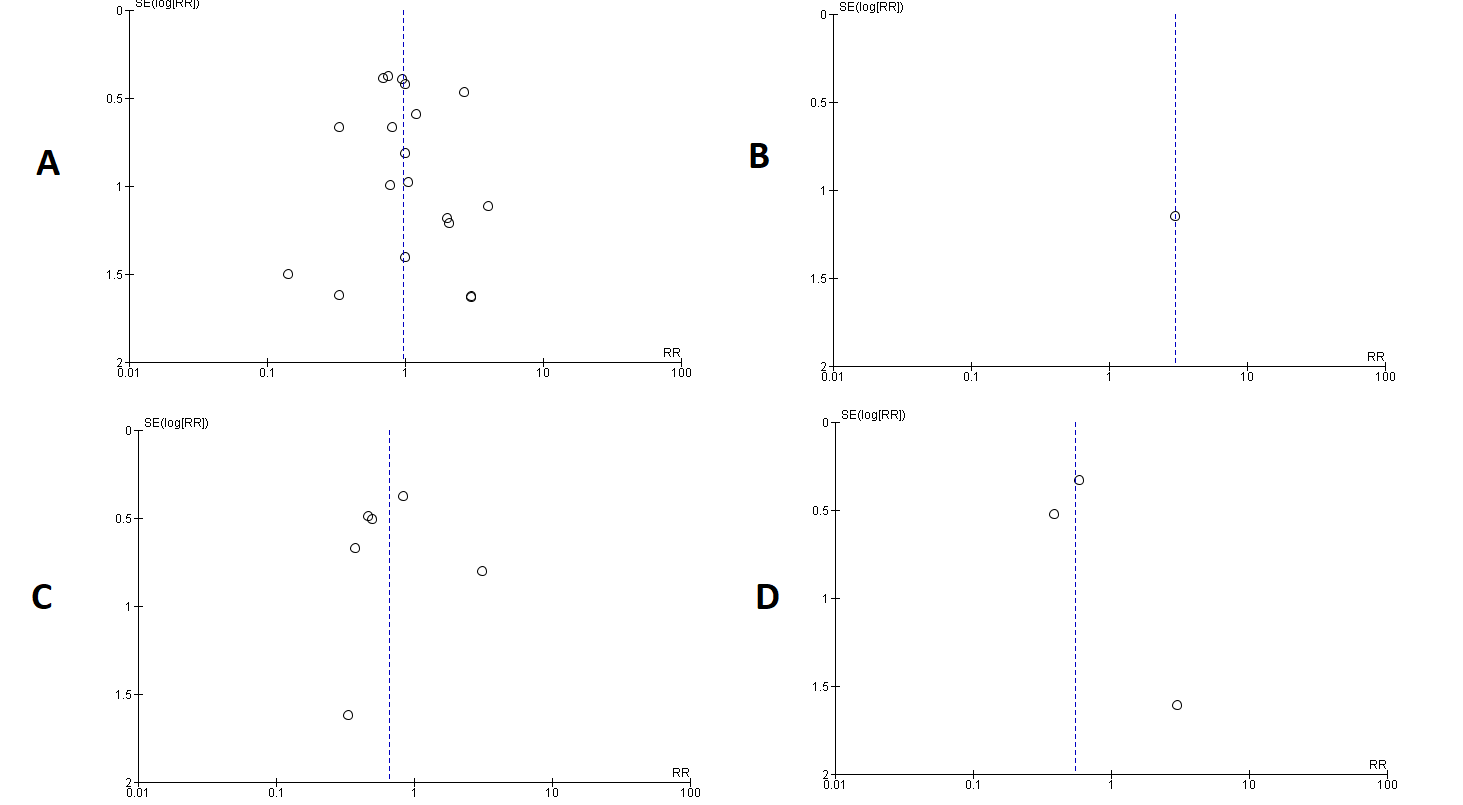


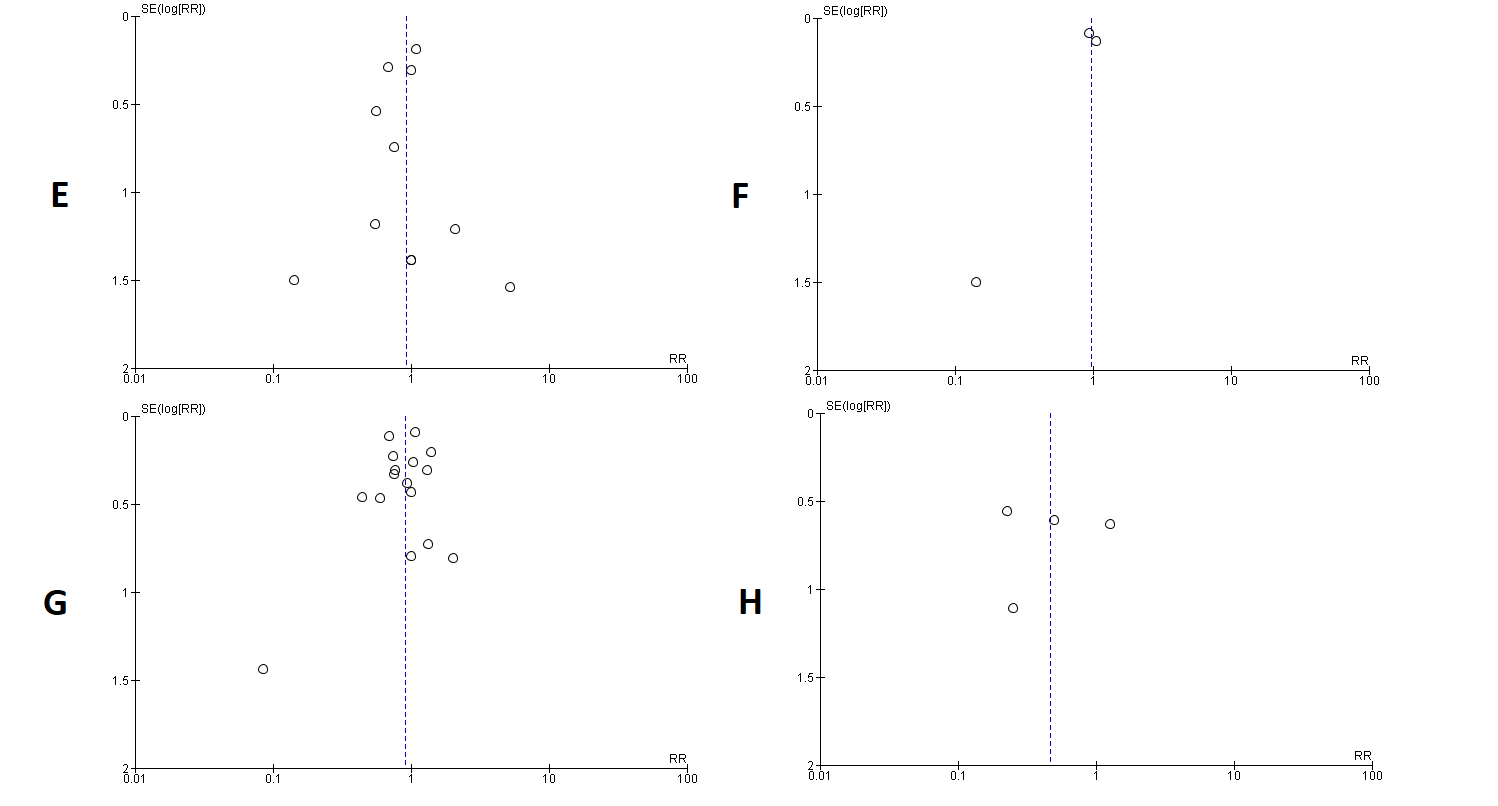


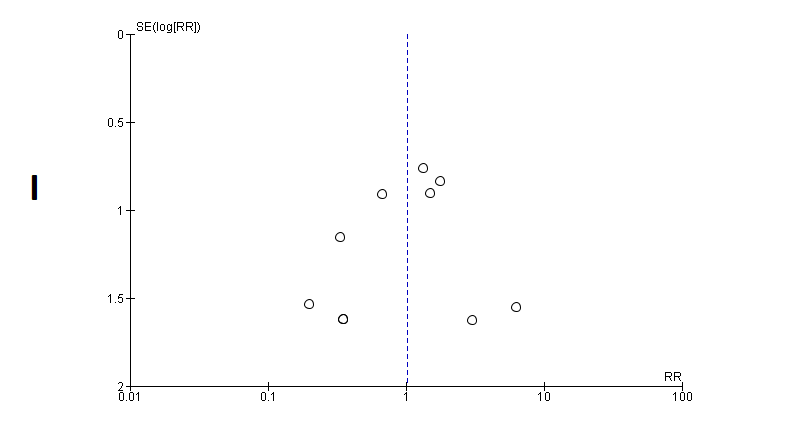


**Figure S5:** Visual funnel plot representation for hematological outcomes in patients undergoing Mini-sternotomy vs Full Sternotomy

A-Red cell transfusion >3

B- Major bleeding

C- Re operation for bleeding

D- Drainage bleed per 24 hrs. in milliliters (ml)

E- Packed Cell Volume (PCV) transfusion

F- paravalvular leak

**[SE: Standard Error; RR: Risk Ratio]**


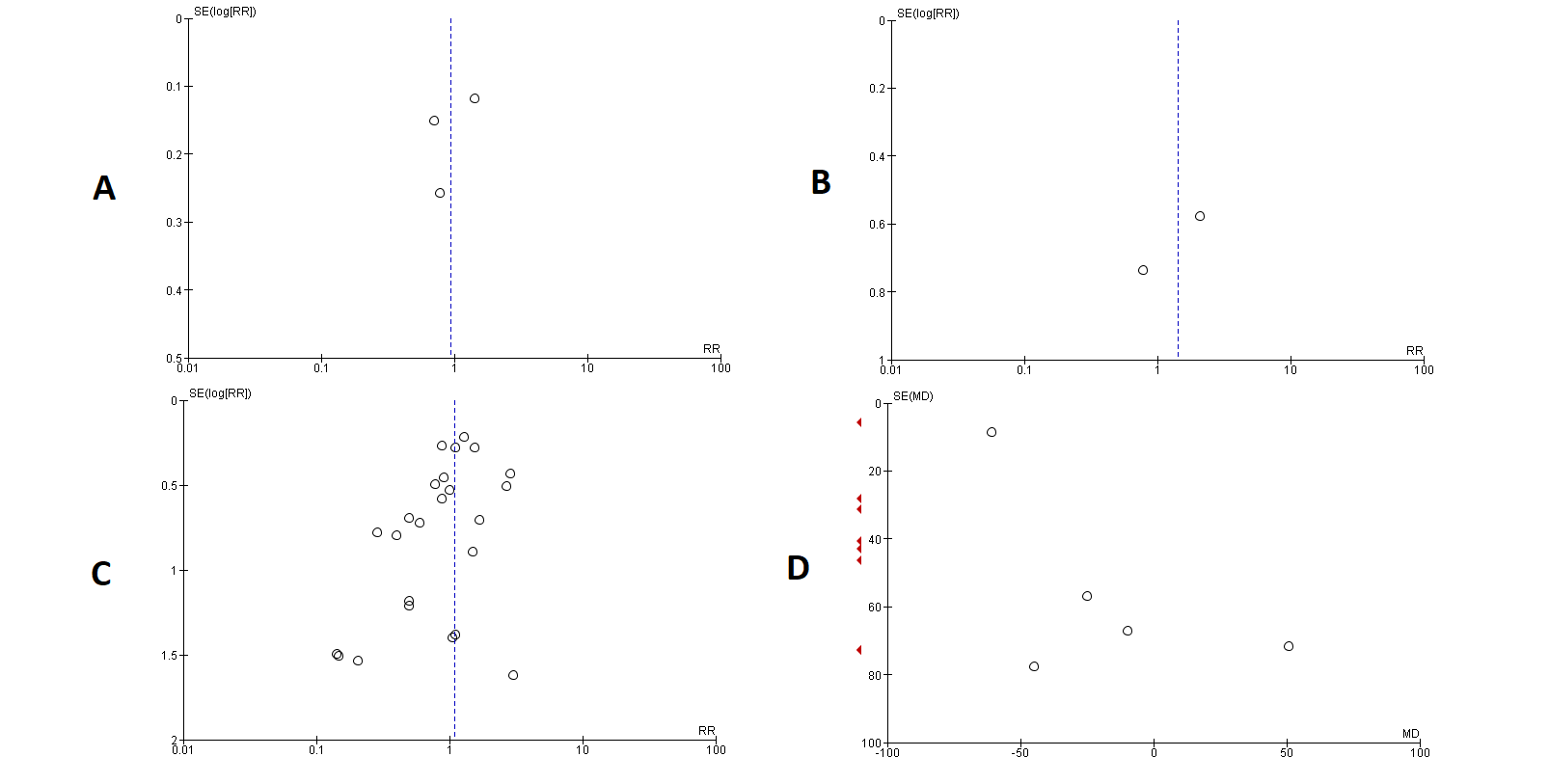


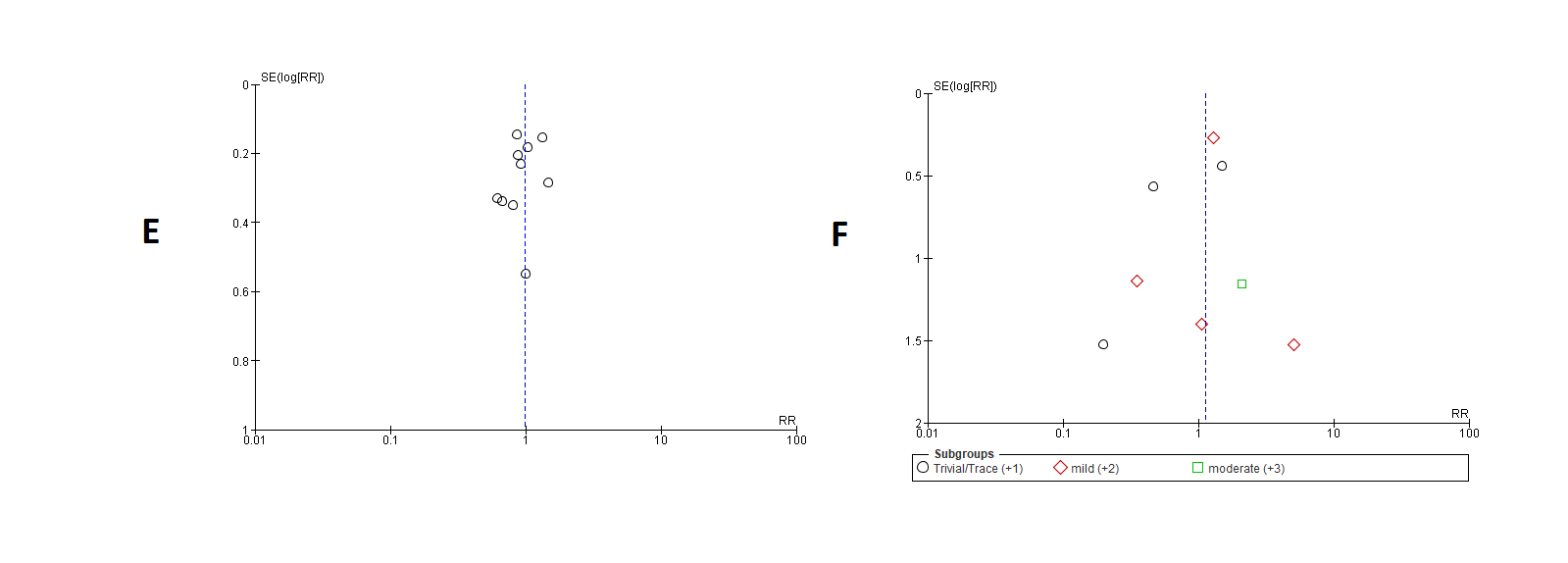


**Figure S6:** Visual funnel plot representation for renal outcomes in patients undergoing Mini-sternotomy vs Full Sternotomy

1. New onset dialysis
2. Renal insufficiency
3. Renal failure

**[SE: Standard Error; RR: Risk Ratio]**


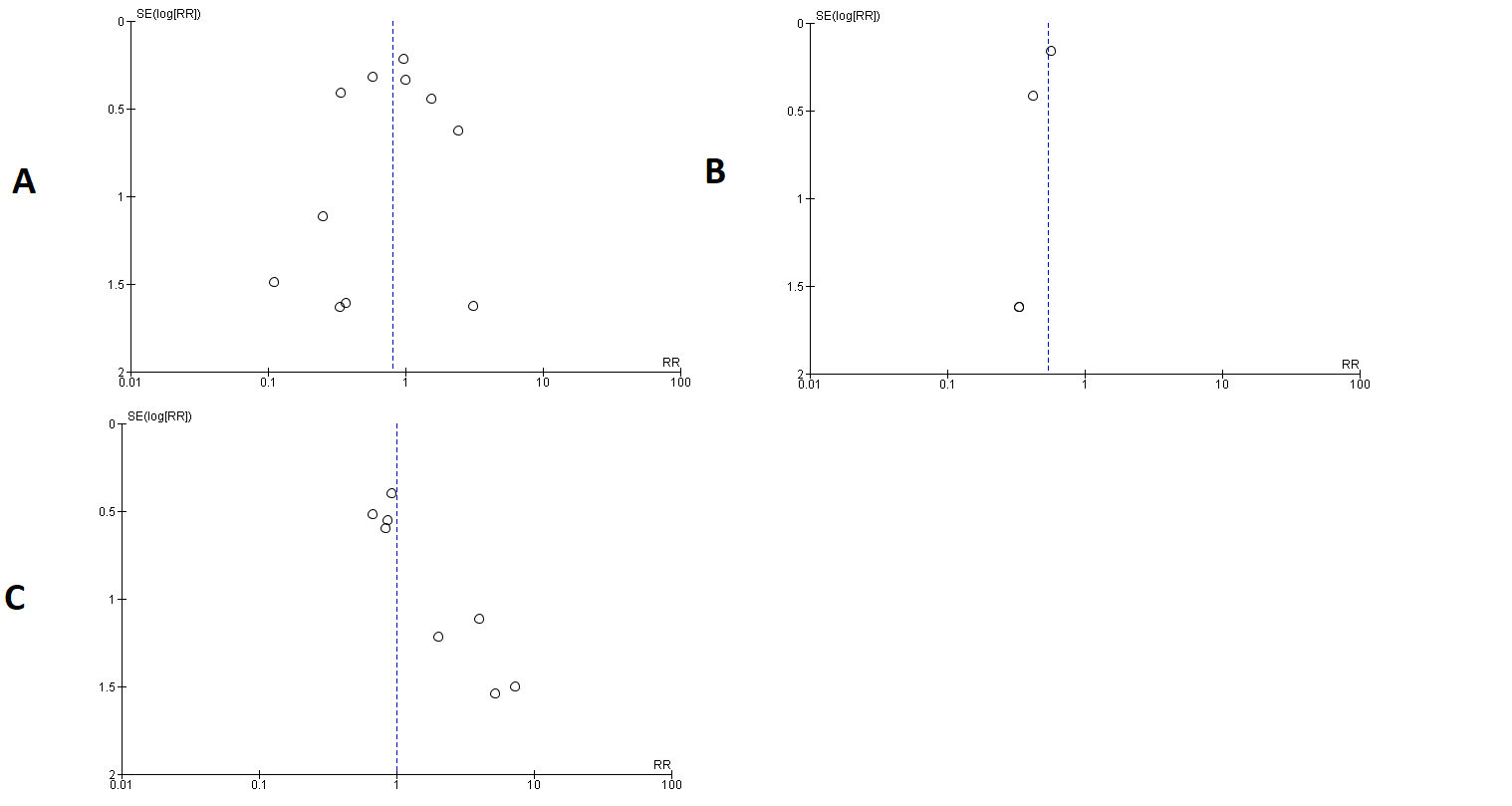


**Figure S7:** Visual funnel plot representation for pulmonary outcomes in patients undergoing Mini-sternotomy vs Full Sternotomy

1. Respiratory insufficiency
2. Respiratory failure
3. Pneumonia

**[SE: Standard Error; RR: Risk Ratio]**


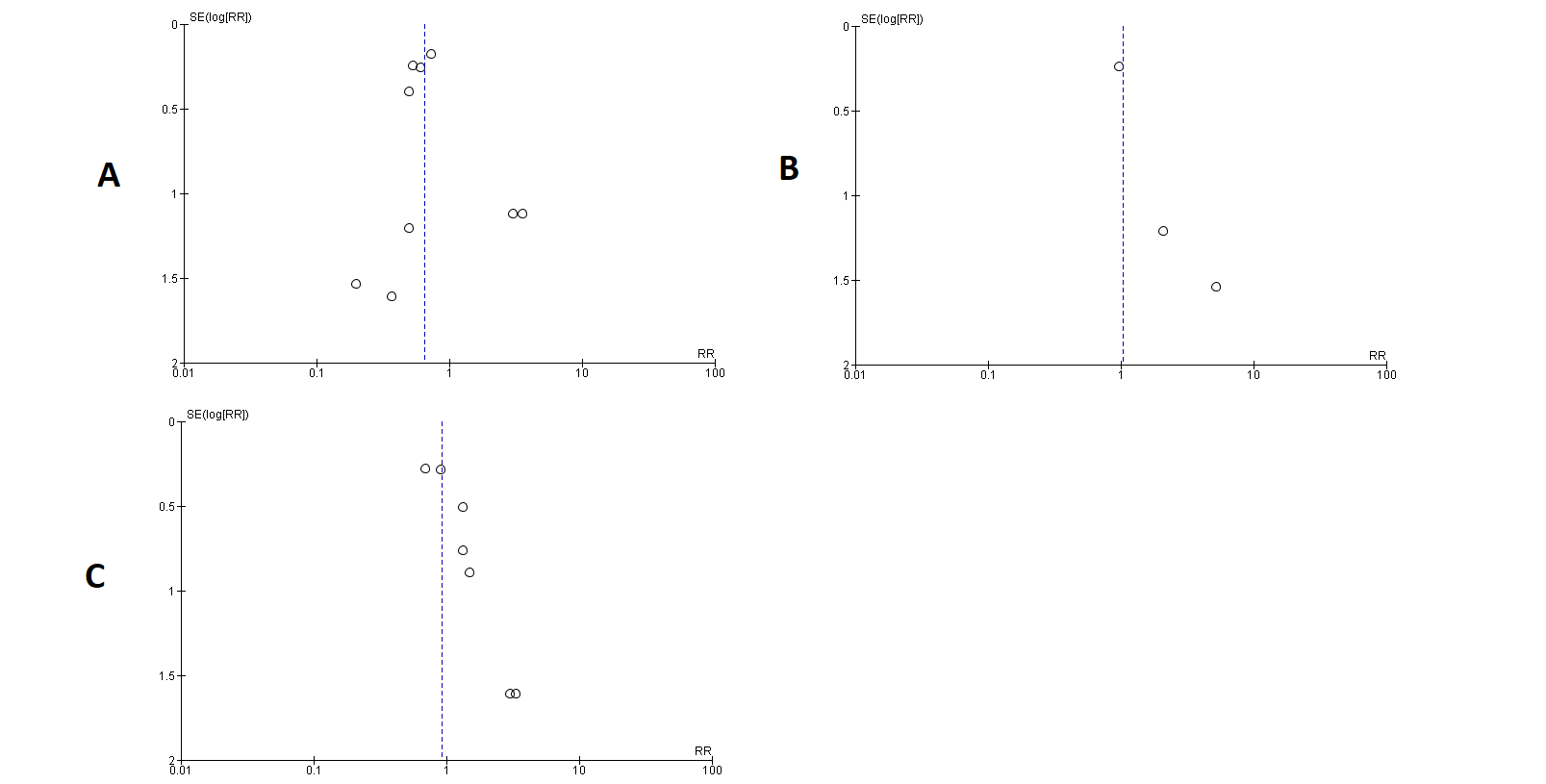


**Figure S8:** Visual funnel plot representation for length of stay (days) in the hospital (A) and the Intensive Care unit (ICU) in patients undergoing Mini-sternotomy vs Full Sternotomy

**[SE: Standard Error; MD: Mean Difference]**


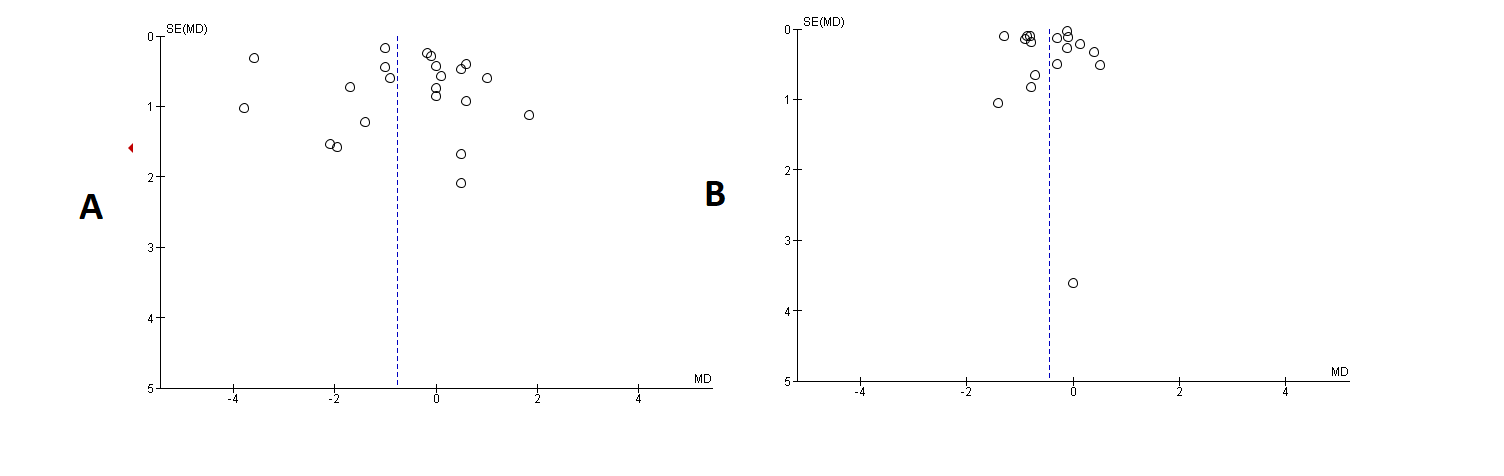


**Figure S9:** Visual funnel plot representation for cross clamp time (A) Cardiopulmonary Bypass (CBP) time (B) and operative time (C) in patients undergoing Ministernotomy vs Full Sternotomy

**[SE: Standard Error; MD: Mean Difference]**


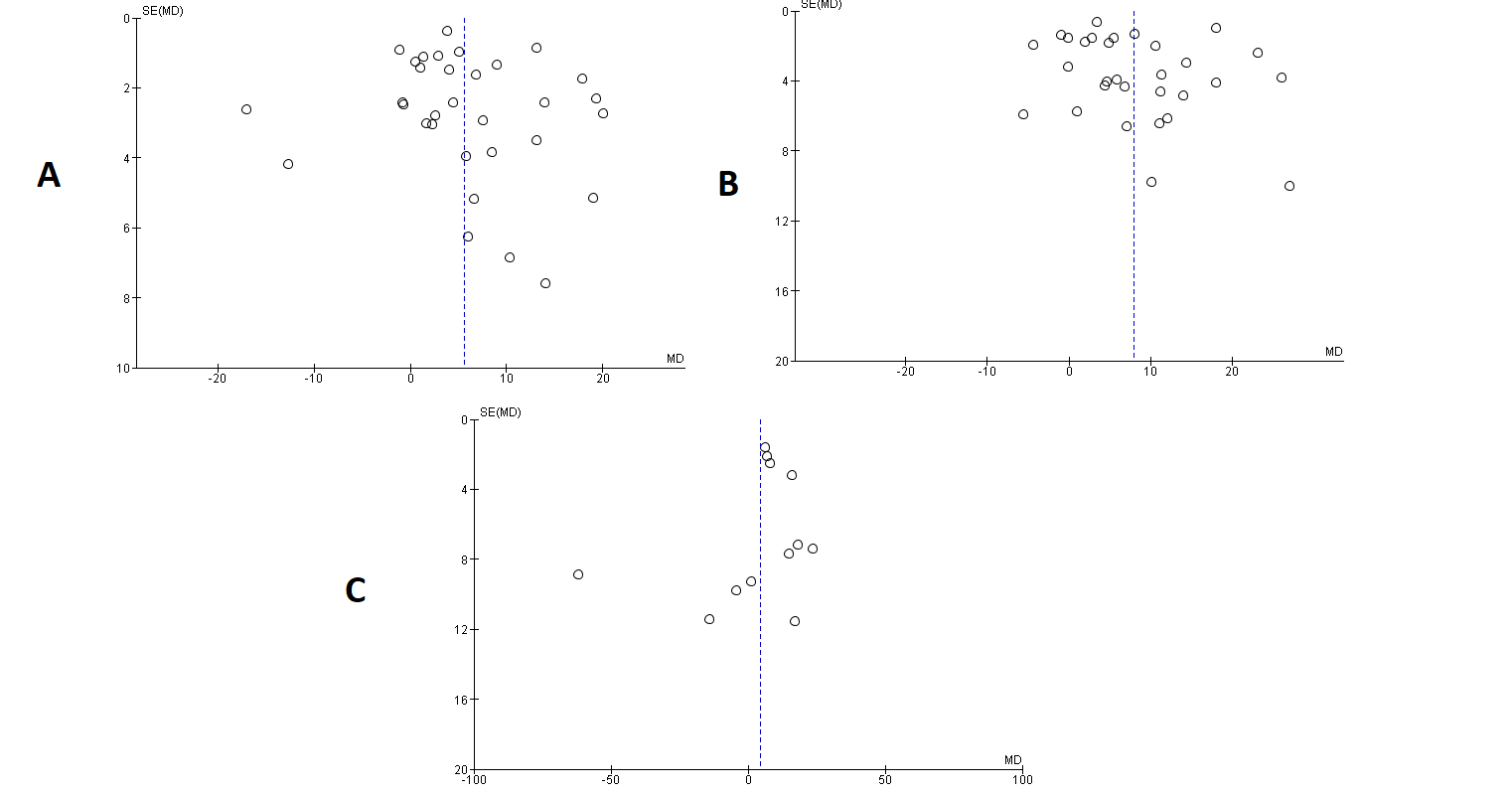


**Figure S10:** Visual funnel plot representation for ventilation in 24 hrs (A) and Extracorporeal Membrane Oxygenation (ECMO) (B) in patients undergoing Ministernotomy vs Full Sternotomy

**[SE: Standard Error; MD: Mean Difference; RR: Risk Ratio]**


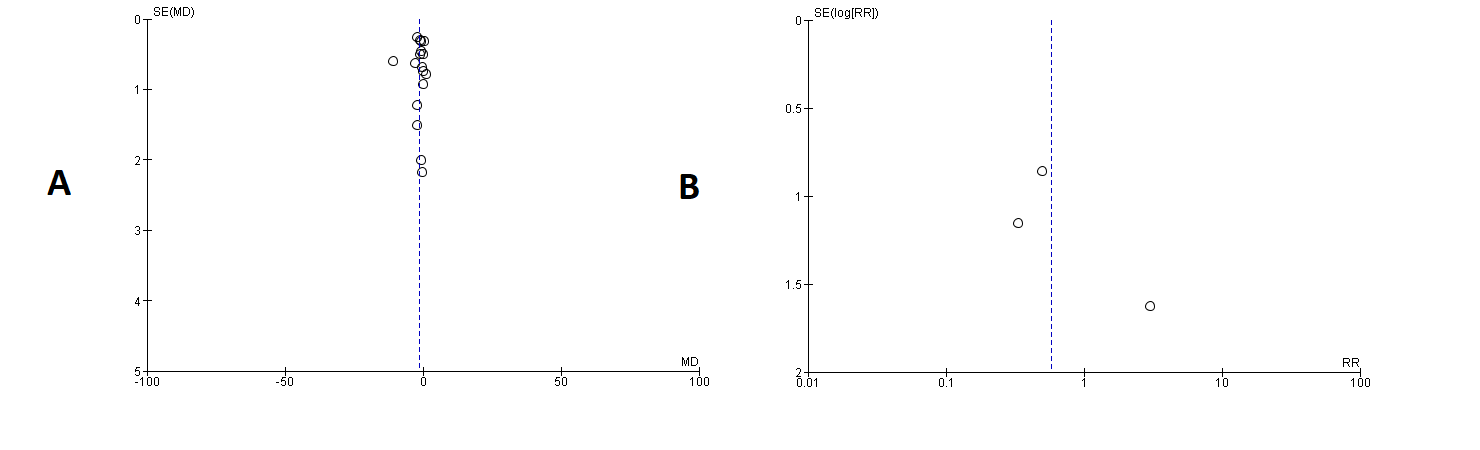


**Figure S11**: Visual funnel plot representation for Surgical Site Infections (SSI) (A) and Sternum Refixation (B) in patients undergoing Ministernotomy vs Full Sternotomy

**[SE: Standard Error; RR: Risk Ratio]**


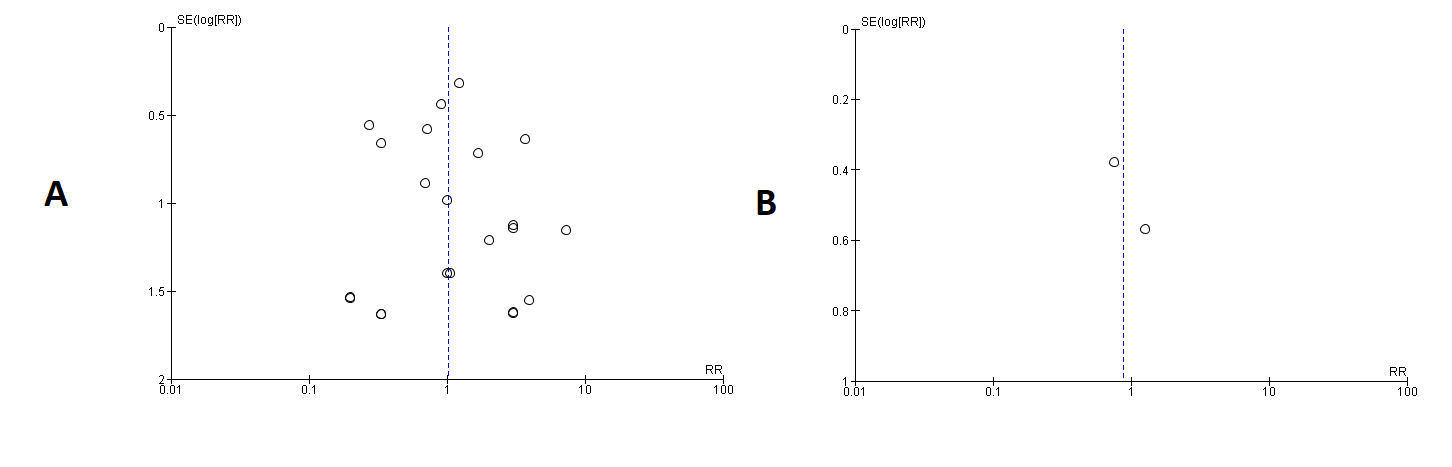


**RMT**

**Figure S12:** Visual funnel plot representation for mortality outcomes in patients undergoing Right Mini Thoracotomy (RMT) vs Full Sternotomy

A-1 year mortality

B -30-day mortality

C- In hospital mortality

**[SE: Standard Error; RR: Risk Ratio]**


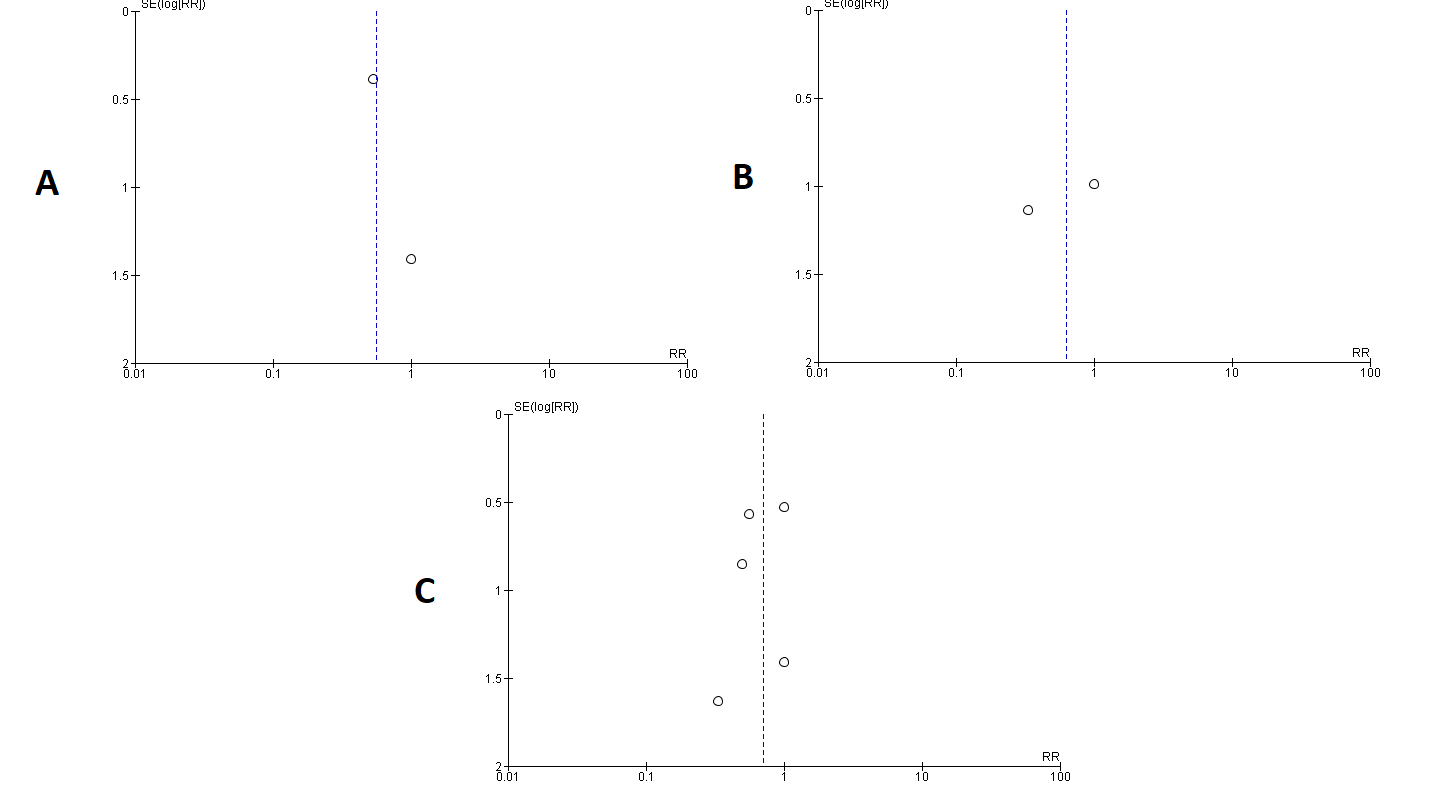


**Figure S13:** Visual funnel plot representation for cardiac outcomes in patients undergoing Right Mini Thoracotomy (RMT) vs Full Sternotomy

A-Stroke

B- Transient Ischemic Attack (TIA)

C-Complete Atrioventricular (AV) Block

D- Permanent Pacemaker

E- Post operative Intra-aortic balloon pump (IABP)

F Post operative Atrial Fibrillation (AF)

**[SE: Standard Error; RR: Risk Ratio]**


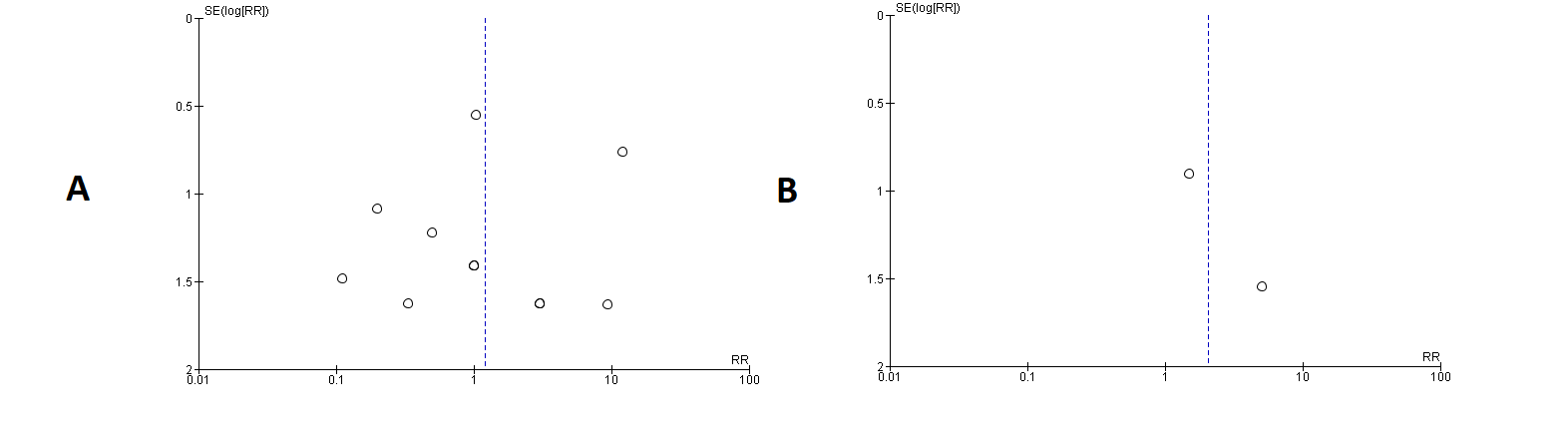


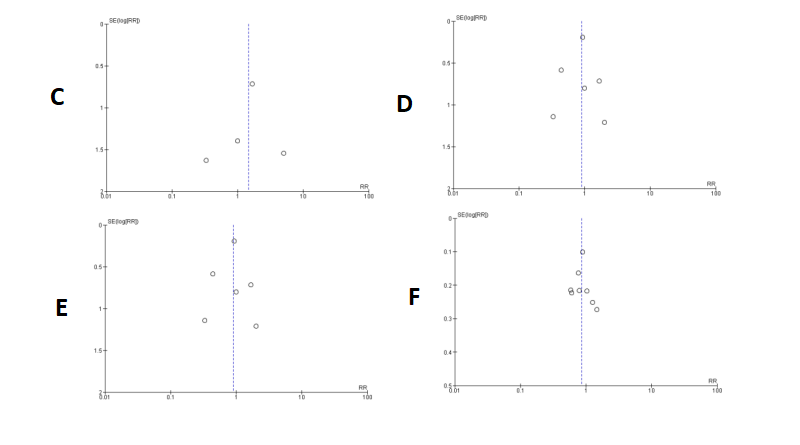


**Figure S14:** Visual funnel plot representation for hematological outcomes in patients undergoing Right Mini Thoracotomy (RMT) vs Full sternotomy

1. Reoperation for bleeding
2. Packed Cell Volume (PCV) transfusion

**[SE: Standard Error; RR: Risk Ratio]**


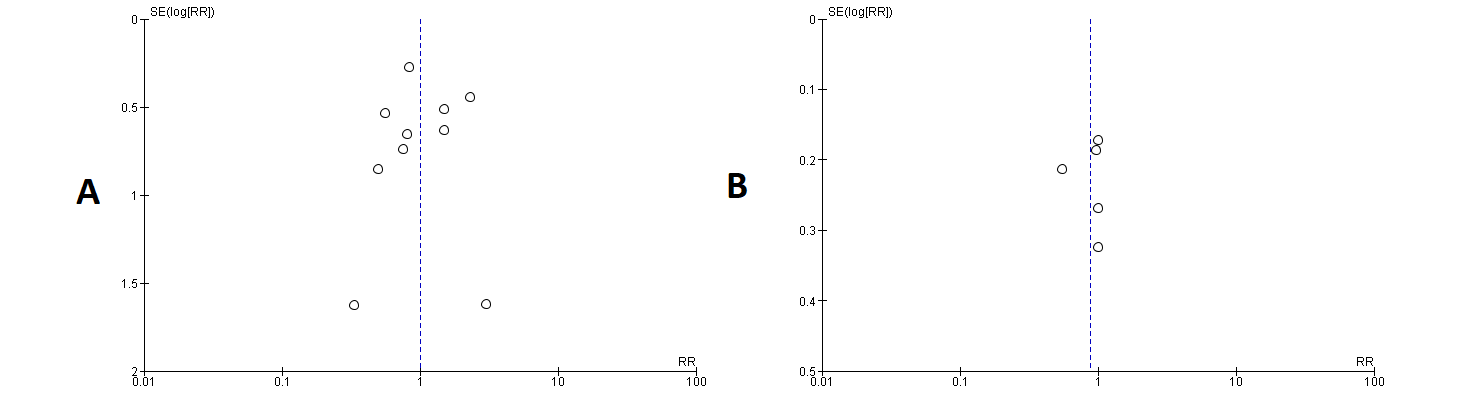


**Figure S15:** Visual funnel plot representation for renal outcomes in patients undergoing Right Mini Thoracotomy (RMT) vs Full Sternotomy

A New onset dialysis

B Renal insufficiency

**[SE: Standard Error; RR: Risk Ratio]**


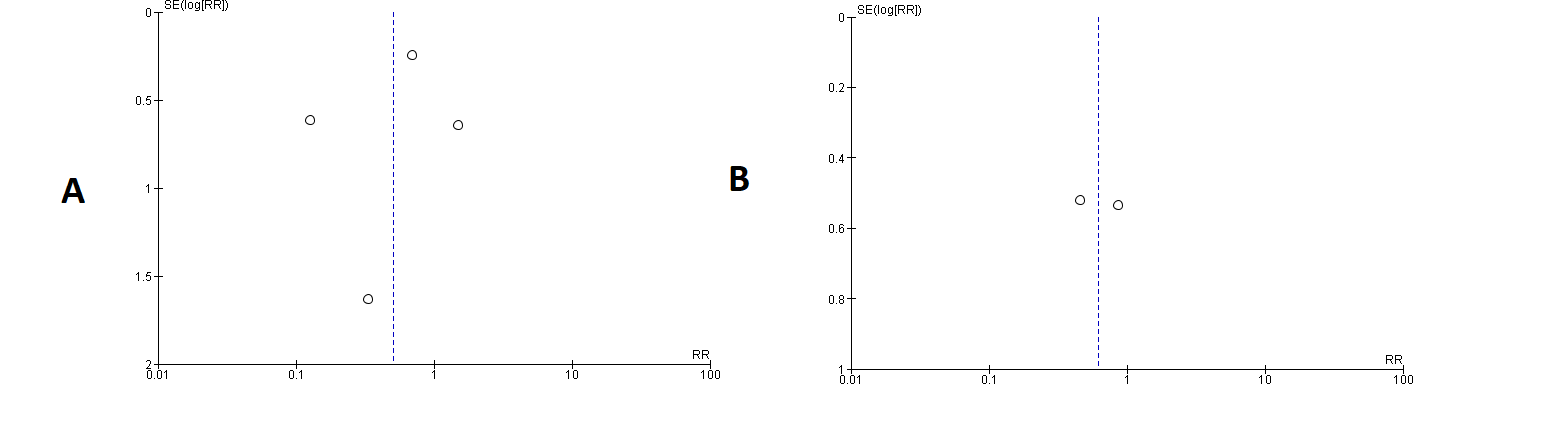


**Figure S16:** Visual funnel plot representation for pulmonary outcomes in patients undergoing Right Mini Thoracotomy (RMT) vs Full Sternotomy

A Respiratory insufficiency

B-Pneumonia

**[SE: Standard Error; RR: Risk Ratio]**


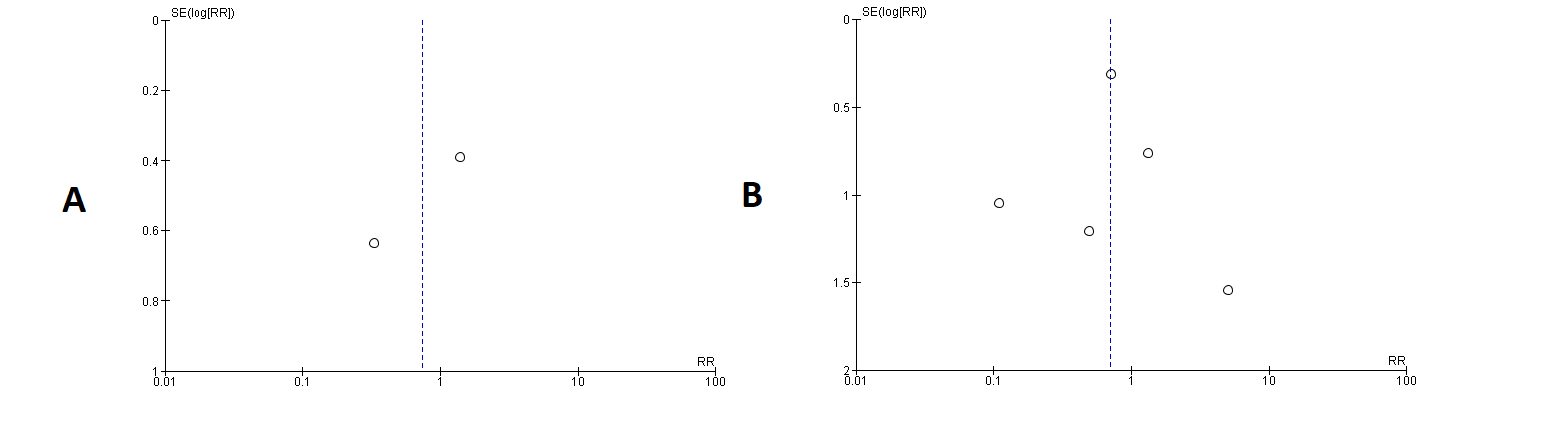


**Figure S17:** Visual funnel plot representation for length of stay (days) in the hospital (A) and the Intensive Care unit (ICU) in patients undergoing Right Mini Thoracotomy (RMT) vs Full Sternotomy

**[SE: Standard Error; Mean Difference]**


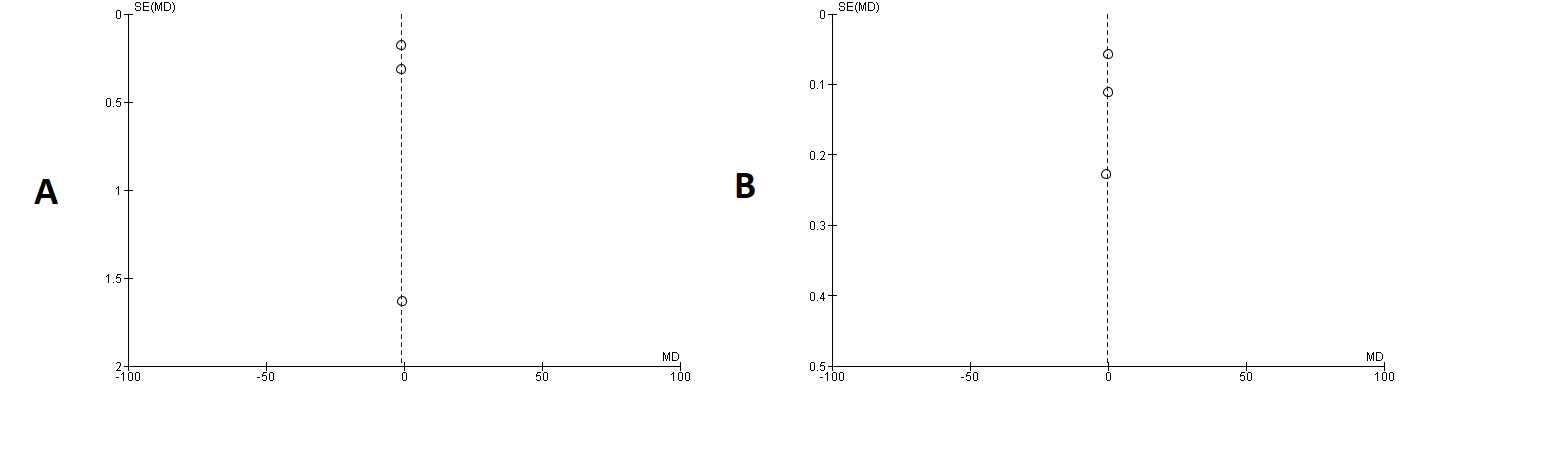


**Figure S18:** Visual funnel plot representation for cross clamp time (A) Cardiopulmonary Bypass (CBP) time (B) and operative time (C) in patients undergoing Right Mini Thoracotomy (RMT) vs Full Sternotomy

**[SE: Standard Error; MD: Mean Difference]**


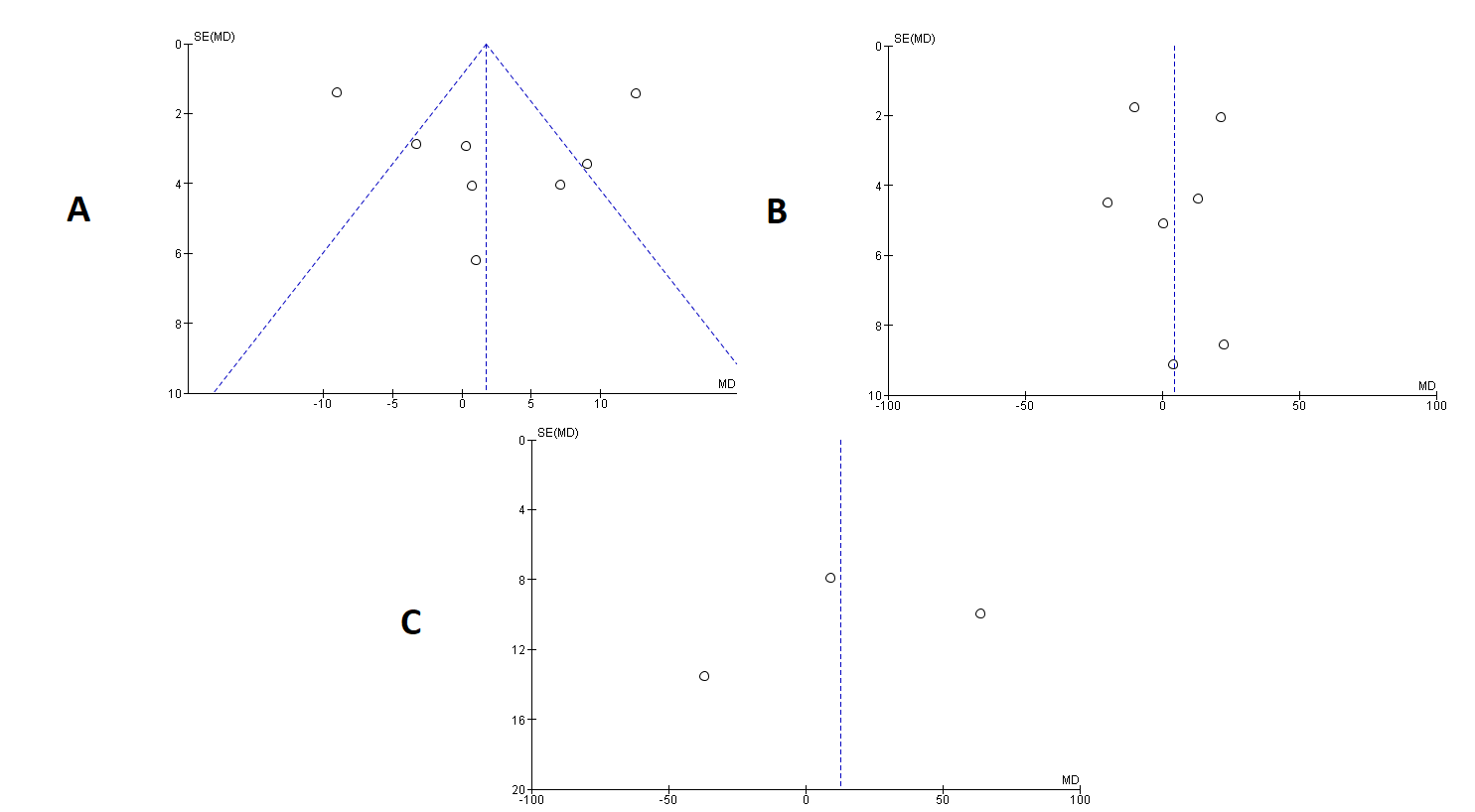


**Figure S19:** Visual funnel plot representation for ventilation in 24 hrs in patients undergoing Right Mini Thoracotomy (RMT) vs Full Sternotomy

**[SE: Standard Error; MD: Mean Difference]**


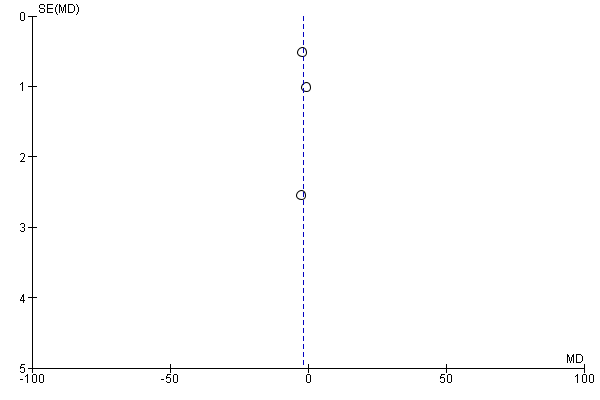


**Forest plots**


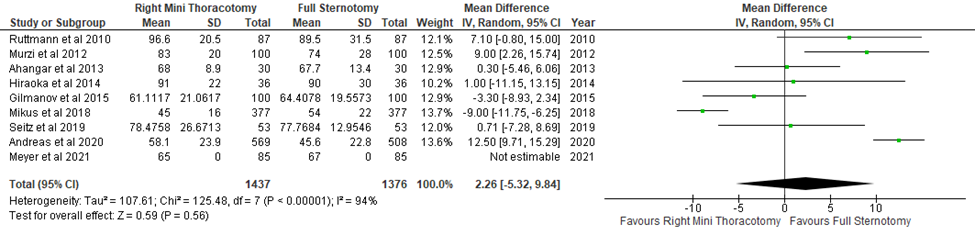


**Figure S20**: Cross Clamp time in patients undergoing Right Mini Thoracotomy (RMT) vs Full Sternotomy

[CI: Confidence Interval; IV: Inverse Variance; SD: Standard Deviation]


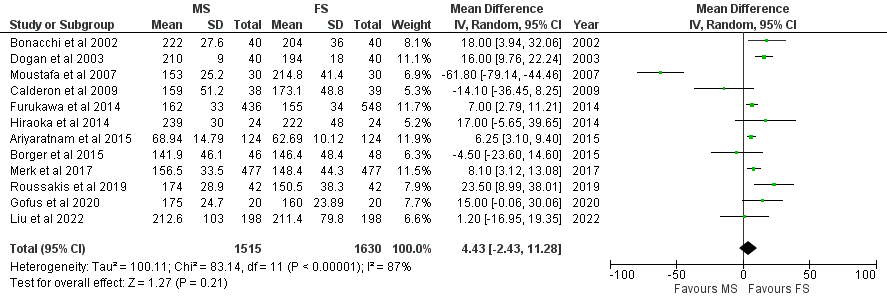


**Figure S21**: Operative time in patients undergoing mini-sternotomy vs full sternotomy

[CI: Confidence Interval; IV: Inverse Variance; SD: Standard Deviation]


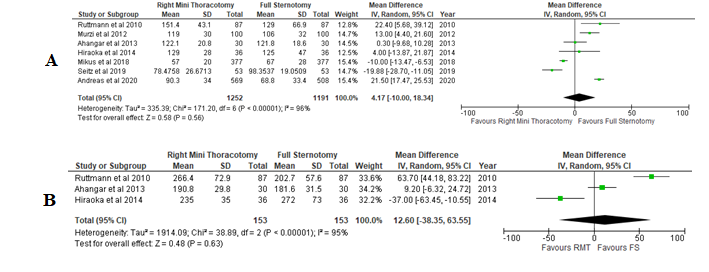


**Figure S22:** (A) Cardiopulmonary Bypass (CPB) time in patients undergoing Right Mini Thoracotomy (RMT) vs Full Sternotomy (B) Operative time in patients undergoing Right Mini Thoracotomy (RMT) vs Full Sternotomy

[CI: Confidence Interval; IV: Inverse Variance; SD: Standard Deviation]


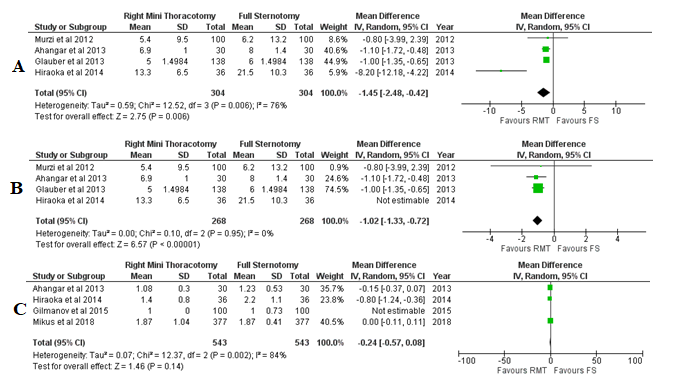


**Figure S23:** (A) Hospital length of stay (days) (B) Leave one out meta-analysis on the outcome of hospital length of stay (C) ICU length of stay in patients undergoing Right Mini Thoracotomy (RMT) vs Full Sternotomy

[CI: Confidence Interval; IV: Inverse Variance; SD: Standard Deviation]


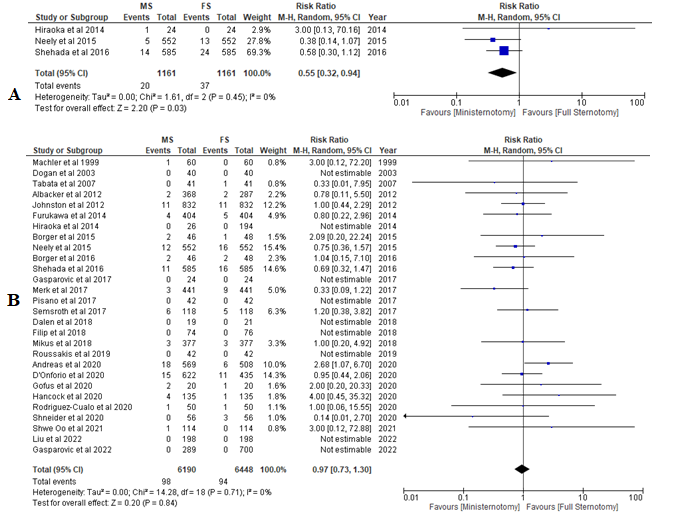

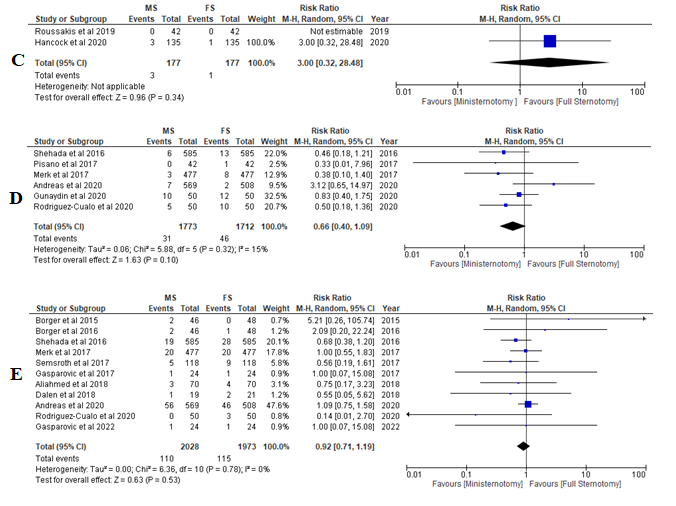

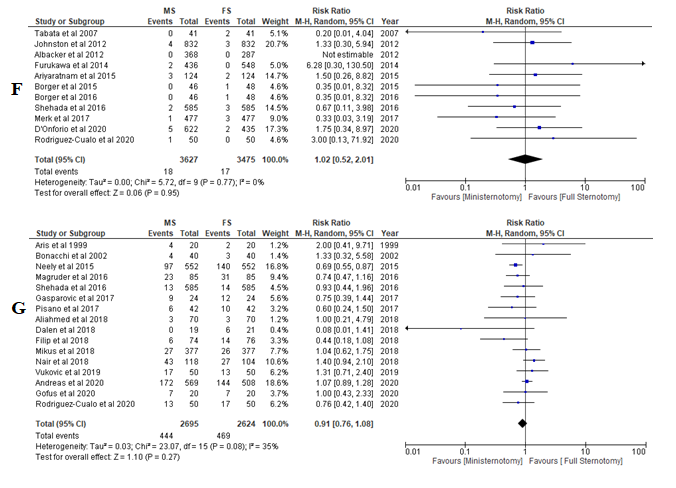


**
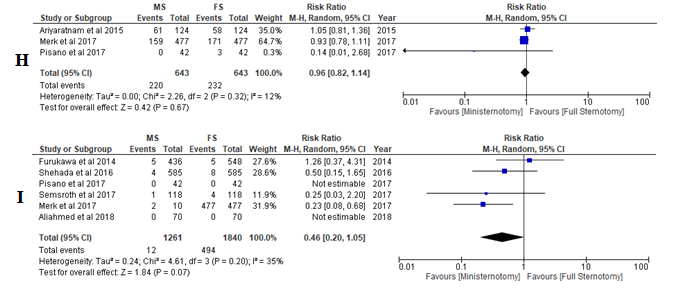
**

**Figure S24:** (A) Complete Atrioventricular (AV) block events (B) Stroke outcomes (C) Transient Ischemic Stroke (TIA) outcomes (D) Low cardiac output (E) permanent pacemaker (F) post operative myocardial infarction (G) post operative atrial fibrillation (H) post operative arrhythmia (I) Post operative Intra-aortic balloon pump (IABP) insertion in patients undergoing Mini-sternotomy vs Full Sternotomy

[CI: Confidence Interval; M-H: Mantel-Haenszel; MS: Mini-sternotomy; FS: Full Sternotomy]


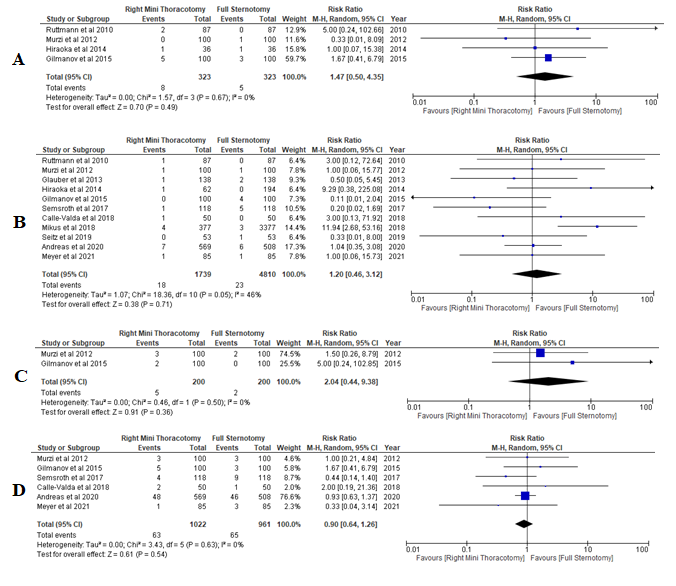

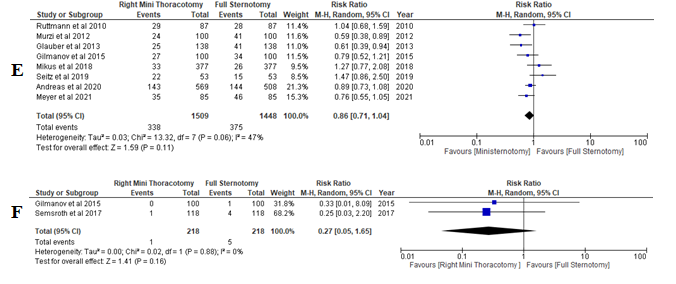


**Figure S25:** (A) Complete Atrioventricular (AV) block events (B) Stroke outcomes (C) Transient Ischemic Stroke (TIA) outcomes (D) permanent pacemaker (E) post operative atrial fibrillation (F) Post operative Intra-aortic balloon pump (IABP) insertion in patients undergoing Right Mini Thoracotomy (RMT) vs Full Sternotomy


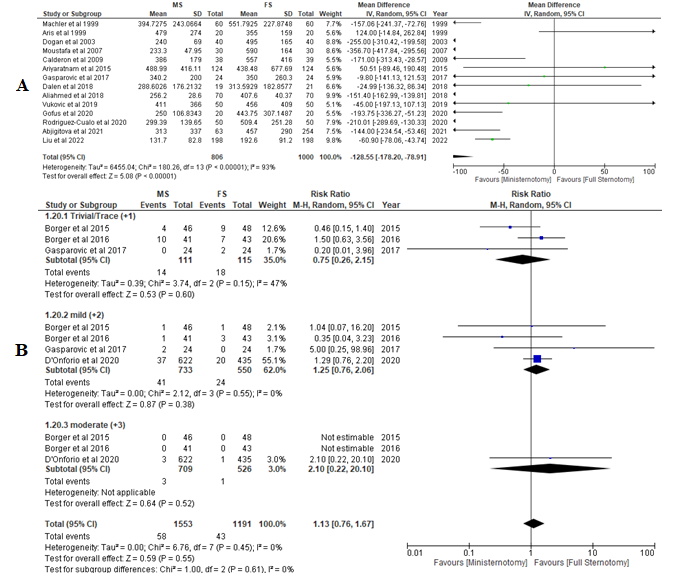

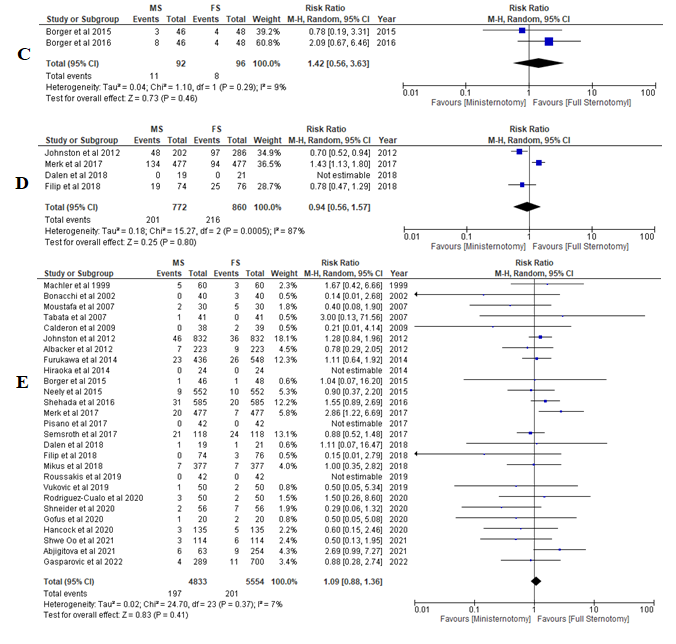

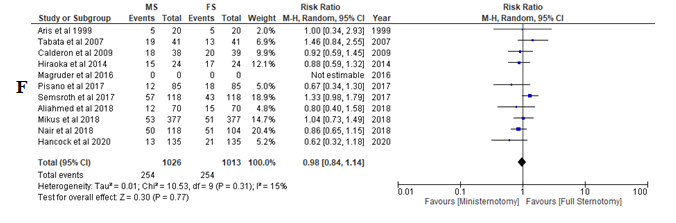


**Figure S26:** (A) Blood drained per 24 hrs in millilitres (ml) (B) Paravalvular leak complications (C) Major bleeding events (D) Red cell transfusion (>3) (E) Reoperation for bleeding (F) Packed cell volume (PCV) transfusion in patients undergoing Mini-sternotomy vs Full Sternotomy


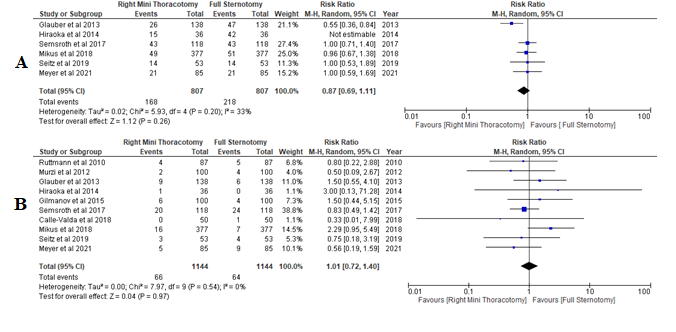


**Figure S27:** (A) Packed cell volume (PCV) transfusion (B) Reoperation for bleeding in patients undergoing Right Mini Thoracotomy (RMT) vs Full Sternotomy


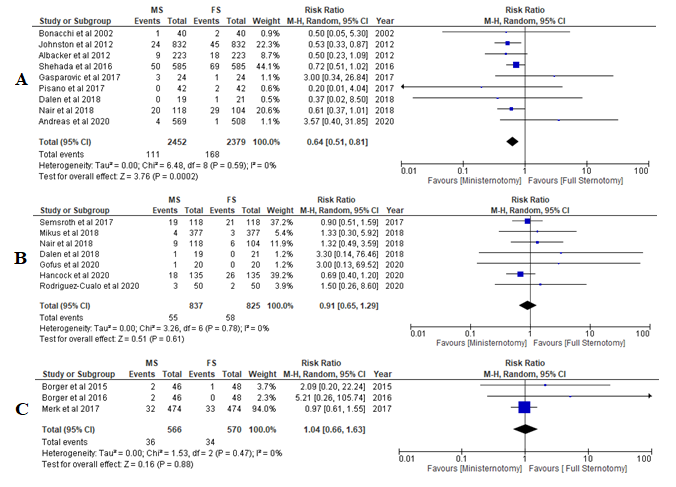

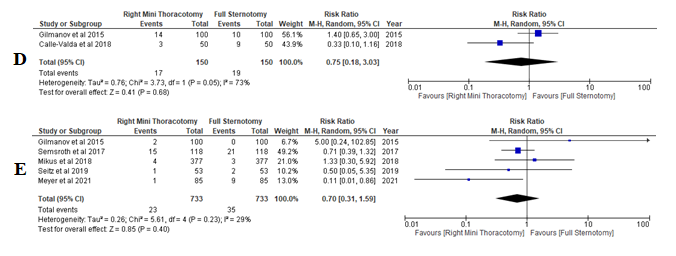


**Figure S28:** (A) Respiratory insufficiency outcomes (B) Pneumonia (C) Respiratory failure events in patients undergoing mini-sternotomy vs Full sternotomy (D) Respiratory insufficiency (E) Pneumonia events in patients undergoing Right mini thoracotomy (RMT) vs full sternotomy


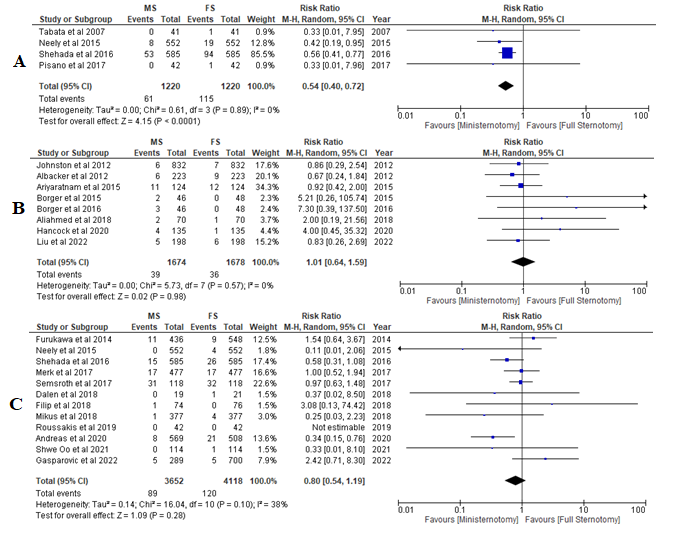


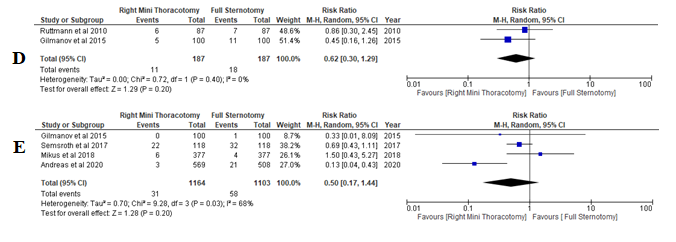


**Figure S29:** (A) Renal insufficiency (B) Renal failure (C) New onset dialysis events in patients undergoing mini-sternotomy vs Full sternotomy (D) Renal insufficiency (E) New onset dialysis events in patients undergoing Right mini thoracotomy (RMT) vs full sternotomy


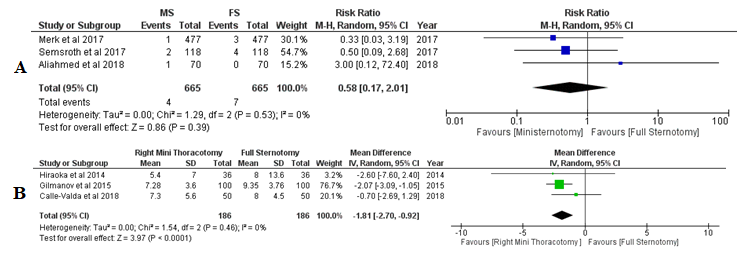


**Figure S30:** (A) Extracorporeal Membrane Oxygenation (ECMO) outcomes in patients undergoing Mini-sternotomy vs Full Sternotomy (B) Ventilation per 24 hours in patients undergoing right mini thoracotomy (RMT) vs full sternotomy


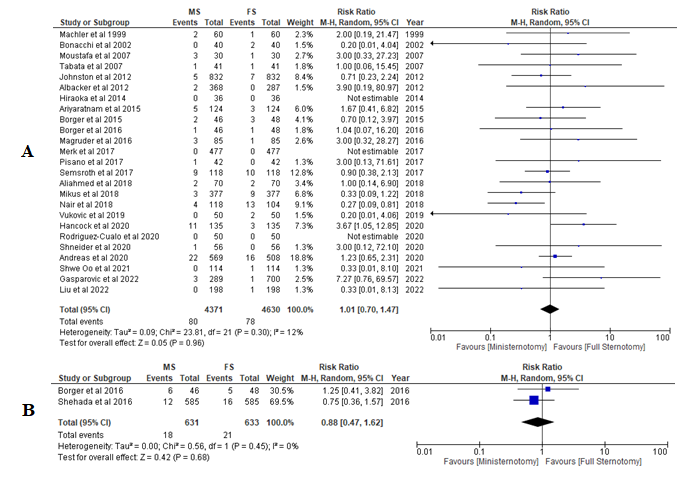


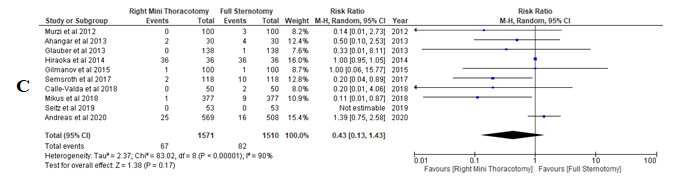


**Figure S31:** (A) Surgical site infections (B) sternum re-fixation in patients undergoing Mini-sternotomy vs Full Sternotomy (C) Surgical site infections in patients undergoing right mini thoracotomy (RMT) vs full sternotomy
